# Efficacy of AAV serotypes to target Schwann cells after intrathecal and intravenous delivery

**DOI:** 10.1038/s41598-021-02694-1

**Published:** 2021-12-02

**Authors:** A. Kagiava, J. Richter, C. Tryfonos, M. Leal-Julià, I. Sargiannidou, C. Christodoulou, A. Bosch, K. A. Kleopa

**Affiliations:** 1 Neuroscience Department, The Cyprus Institute of Neurology and Genetics and Cyprus School of Molecular Medicine, 6 Iroon Avenue, P.O. Box 23462, 1683 Nicosia, Cyprus; 2 Center for Neuromuscular Diseases, The Cyprus Institute of Neurology and Genetics and Cyprus School of Molecular Medicine, Nicosia, Cyprus; 3 Molecular Virology Department, The Cyprus Institute of Neurology and Genetics and Cyprus School of Molecular Medicine, Nicosia, Cyprus; 4 Department of Biochemistry and Molecular Biology, Institute of Neurosciences, Barcelona, Spain; 5 Centro de Investigación Biomédica en Red Sobre Enfermedades Neurodegenerativas (CIBERNED), Bellaterra, Spain; 6 Unitat Mixta UAB-VHIR, Vall d’Hebron Institut de Recerca (VHIR), Barcelona, Spain

**Keywords:** Diseases of the nervous system, Myelin biology and repair, Peripheral nervous system

## Abstract

To optimize gene delivery to myelinating Schwann cells we compared clinically relevant AAV serotypes and injection routes. AAV9 and AAVrh10 vectors expressing either EGFP or the neuropathy-associated gene *GJB1*/Connexin32 (Cx32) under a myelin specific promoter were injected intrathecally or intravenously in wild type and *Gjb1-*null mice, respectively. Vector biodistribution in lumbar roots and sciatic nerves was higher in AAVrh10 injected mice while EGFP and Cx32 expression rates and levels were similar between the two serotypes. A gradient of biodistribution away from the injection site was seen with both intrathecal and intravenous delivery, while similar expression rates were achieved despite higher vector amounts injected intravenously. Quantified immune cells in relevant tissues were similar to non-injected littermates. Overall, AAV9 and AAVrh10 efficiently transduce Schwann cells throughout the peripheral nervous system with both clinically relevant routes of administration, although AAV9 and intrathecal injection may offer a more efficient approach for treating demyelinating neuropathies.

## Introduction

Inherited neuropathies known as Charcot-Marie-Tooth (CMT) disease are heterogeneous disorders caused by mutations in many different genes and remain without effective treatment^1,2^. The demyelinating CMT forms are the most common and result from mutations in genes that are highly expressed in myelinating Schwann cells having mostly cell-autonomous mechanisms^1,3^. As gene therapy approaches are emerging as potential treatment for neuromuscular disorders^4–8^, the main challenge for their application in demyelinating CMT neuropathies is the need to efficiently target Schwann cells throughout the peripheral nervous system (PNS). The aim of this study was to clarify the most efficient viral vectors and clinically relevant delivery methods to specifically target PNS myelinating Schwann cells.

Adeno-associated viral (AAVs) vectors have been established as a very useful tool for gene delivery mostly into the CNS for the treatment of neurological disorders. In particular AAV9 is the most commonly used serotype with a demonstrated tropism towards the CNS and especially for motor neurons when combined with the CBh or the CBA promoters^9,10^. AAV9 carrying the gigaxonin gene has been tested for treatment of Giant Axonal Neuropathy (GAN)^11^ and also provides expression of the *SMN1* gene^12^ currently used by intravenous or intrathecal delivery for treatment of Spinal Muscular Atrophy (SMA). In addition, spinal subpial delivery of AAV9 carrying an shRNA prevents spinal cord atrophy in the SOD1(G93A) mouse model of familial amyotrophic lateral sclerosis (FALS)^13^.

AAVrh10 is another commonly used serotype for gene delivery in different disease models including the twicher model for Krabbe disease^14,15^, in large animals^16^, and in diabetic neuropathy models^17^. AAVrh10 has shown expression in the PNS including dorsal root ganglia (DRGs)^17^ and spinal nerves^16^, with functional^17^ and morphological improvements in peripheral nerves^14,15^. Although these studies showed that the genes carried by AAVrh10 can be expressed in the PNS, expression was driven by ubiquitous promoters and was not targeted to Schwann cells. Thus, improvement observed could result from cross-correction through the transfer of the protein from axons and other cells surrounding the PNS.

Among different routes of administration of viral vectors to treat neurological disorders, the two most clinically relevant and least invasive are the intravenous and the intrathecal injections. Intravenous injection is the most applicable to humans but has significant disadvantages including high vector amount needed for injection in order to achieve expression in the CNS and the immunogenicity caused by pre-existing antibodies in most humans^18–22^. Delivery of the virus into the cisterna magna has been the most efficient way for targeting CNS^23–25^ but has limited usefulness in clinical practice due to the invasiveness and increased risk.

Lumbar intrathecal delivery could be the safest and most effective route of administration to target both CNS and PNS as it has been show to result in widespread expression of both AAV9^23,26,27^ and AAVrh10^14,16,17,28^ although recent data indicate that it may not achieve the necessary CNS biodistribution for translation in humans^29^. Studies are mainly focused on the CNS biodistribution while there are data showing that both vectors can reach the sciatic nerve^14,16,17,23^ although with no targeted expression.

Here we compared the efficacy of AAV9 and AAVrh10 to transduce Schwann cells in PNS tissues using the Schwann cell-specific rat Mpz promoter, as well as the two most clinically relevant routes of administration, intravenous and intrathecal injection. AAV9 and AAVrh10 vectors expressing either the reporter gene EGFP or the neuropathy-associated gene *GJB1* were injected into adult wild type (WT) or *Gjb1*-null mice, respectively. The expression of EGFP and Cx32 was assessed by immunostaining and immunoblot analysis. Both serotypes showed similar biodistribution into the PNS when using the same route of injection. However, AAV9 showed a slightly better capsid/expression ratio. Furthermore, both routes of administration proved to be equally efficient for Schwann cell-targeted gene delivery throughout the PNS, with the advantage of the intrathecal injection requiring much smaller vector amounts and being less immunogenic. Our results indicate that both clinically relevant AAV serotypes could be used for the treatment of demyelinating peripheral neuropathies and that lumbar intrathecal injection might be the most preferable route of administration.

## Results

### Vector biodistribution following intrathecal and intravenous injection of AAV9 and AAVrh10 vectors targeting Schwann cells

AAV9 and AAVrh10 vectors were produced at high titers of 1 × 10^13^ vg/ml and delivered either by intravenous injection of 1 × 10^12^ vg per mouse or by lumbar intrathecal injection of 2 × 10^11^ vg per mouse. AAV9-*Mpz*.*Egfp* and AAVrh10-*Mpz*.*Egfp* vectors carrying the *Egfp* gene were injected into 2-month-old WT mice, while AAV9-*Mpz*.*GJB1* and AAVrh10- *Mpz*.*GJB1*vectors carrying the *GJB1* gene were injected in *Gjb1*-null mice in order to study their biodistribution and compare their efficacy to transduce Schwann cells. VGCNs were measured 6 weeks post-injection in relevant PNS and CNS tissues.

**Figure 1:**
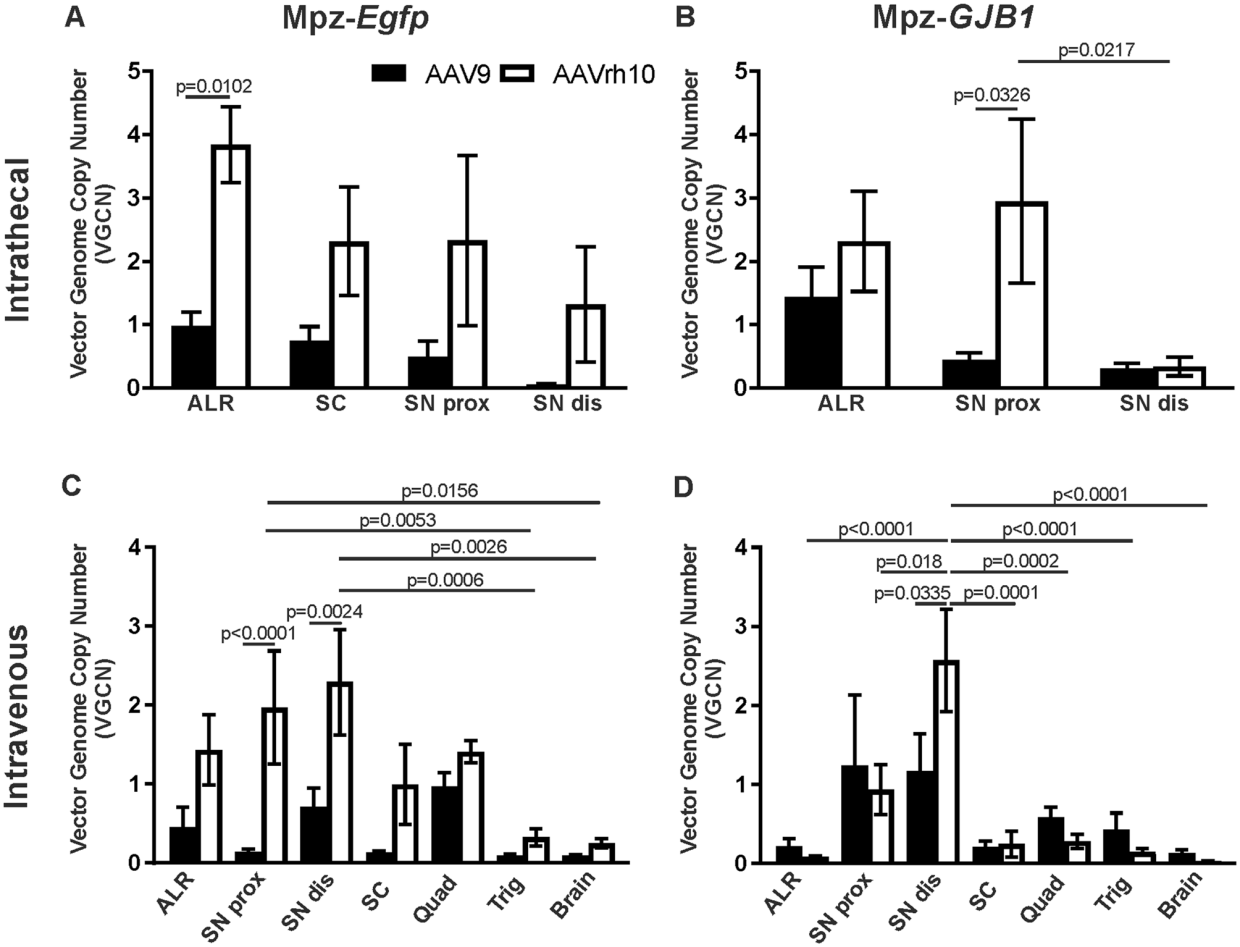
Biodistribution of AAV9 and AAVrh10 in different tissues following intravenous and intrathecal injection. Determination of the vector genome copy numbers (VGCN) in different tissues after intrathecal (**A-B**) or intravenous (**C-D**) injection of AAV9 and AAVrh10 vectors carrying the *Egfp* and the *GJB1* gene driven by the myelin protein zero (Mpz) promoter. VGCNs of AAVrh10 were higher in most of the tissues examined using both serotypes with either Mpz.*Egfp* or Mpz.*GJB1* expression constructs. Biodistribution data show that intravenous injection targets more efficiently distal PNS tissues while intrathecal injection targets both the CNS and proximal more than distal PNS tissues with a gradient pattern. AAVrh10 biodistribution is generally higher compared to AAV9 in most tissues (data are shown in Tables 1 and 2). (ALR: anterior lumbar root; SN: sciatic nerves; prox: proximal; dis: distal; SC: spinal cord; quad: quadriceps muscle; trig: trigeminal nerve). Two-way ANOVA revealed a prevalence of AAVrh10 to target sciatic nerves after intravenous injection with the Mpz.*Egfp* vector (F(1, 65) = 27.71, *p* < 0.0001) and a gradient from nerves to the CNS only in the case of AAVrh10 (F(6, 65) = 5.338, *p* = 0.0002). Tukey’s posthoc testing showed differences between SN dis and brain (adj. *p* = 0.0026), SN dis and trigeminal (adj. *p* = 0.0006), SN prox and brain (adj. *p* = 0.0156) and SN prox and trigeminal (adj. *p* = 0.0053). Finally, AAVrh10 VGCNs were higher in the SN dis and SN prox compared to AAV9 (adj. *p* = 0.0024 and *p* < 0.0001 respectively). Intrathecal injection of the same vector revealed only a higher biodistribution of AAvrh10 in ALR (F(1, 57) = 16.59, *p* = 0.0001). Tukey’s posthoc test showed significant difference between the two serotypes in ALR (adj. *p* = 0.0102). In the case of Mpz.*GJB1* vector following intravenous injection two-way ANOVA showed again a gradient with higher VGCNs closer to the injection site and lower distally from the injection site and only for AAVrh10 (F(6, 70) = 7.436, *p* < 0.0001) and with higher AAvrh10 biodistribution only for the SN dis (F(1, 70) = 0.05926, *p* = 0.8084). Tukey’s posthoc testing showed differences between SN dis and all the other tissues examined (ALR: adj. *p* < 0.0001, SN prox: adj. *p* = 0.018, SC: adj. *p* = 0.0001, Brain: adj. *p* < 0.0001, Quad: adj. *p* = 0.0002, Trig: adj. *p* < 0.0001). Finally, AAVrh10 VGCNs were higher in the SN dis compared to AAV9 (adj. *p* = 0.0335). Intrathecal injection revealed a higher biodistribution of AAvrh10 compared to AAV9 in the SN prox (F(1, 30) = 4.56, *p* = 0.0401) and a gradient from the SN prox to SN dis (F(2, 30) = 3.405, *p* = 0.0465) Tukey’s posthoc showed statistical difference between the two serotypes in the proximal part of the nerve (adj. *p* = 0.0326) and in the case of AAVrh10 there were higher VGCNs in the proximal part (adj. *p* = 0.0217). Data are represented as mean ± SEM. Data of the two vectors and different tissues were compared using 2-way ANOVA with Tukey’s post-test.

In WT mice injected intrathecally with the AAV9 or AAVrh10 (Fig. 1A; Table 1) higher VGCNs were obtained with AAVrh10 (range from 3.84 ± 0.6 in lumbar roots to 1.32 ± 0.91 in distal sciatic nerve) compared to AAV9 (range 0.98 ± 0.23 in lumbar roots to 0.06 ± 0.01 in distal sciatic nerve), but this was only statistically significant in the lumbar roots (*p* = 0.0102). As expected, a gradient of decreasing biodistribution was noted with both vectors from the spinal roots to proximal and distal sections of the sciatic nerves, although without any statistical difference. Both vectors were also detected in the spinal cord with AAVrh10 again showing higher VGCNs (2.32 ± 0.86) compared to AAV9 (0.75 ± 0.22 although not statistically significant due to variation). Similar analysis following intrathecal injection of vectors driving *GJB1* expression in *Gjb1*-null mice (Fig. 1B; Table 1) showed again higher VGCNs in AAVrh10-injected mice (range 2.32 ± 0.79 in lumbar roots to 0.34 ± 0.15 in distal nerves) compared to AAV9 (range 1.44 ± 0.47 in lumbar roots to 0.31 ± 0.08 in distal nerves) and a gradient from proximal to distal PNS tissues, especially in the case of AAVrh10 where we observed a statistically significant gradient from the proximal towards the distal sciatic nerve (*p* = 0.0217). Overall, intrathecal injection results in a gradient of biodistribution which is mostly higher with AAVrh10 compared to AAV9 vectors.

**Table 1:** Vector genome copy numbers (VGCN) in different tissues of the PNS and spinal cord of WT mice injected intrathecally with AAV9-Mpz.*Egfp* or AAVrh10-Mpz.*Egfp* and of *Gjb1*-null mice injected intrathecally with AAV9-Mpz.*GJB1* or AAVrh10-Mpz.*GJB1.* WT: wild type; ND: not done; The values represent mean ± SEM.

Tissue	WT mice			*Gjb1-null mice*		
	AAV9-Mpz.*Egfp*	AAVrh10-Mpz.*Egfp*	*P* value	AAV9-Mpz.GJB1	AAVrh10-Mpz.*GJB1*	*P* value
Lumbar roots	0.98 ± 0.23 (n = 10)	3.84 ± 0.60 (n = 7)	0.0102	1.44 ± 0.47 (n = 6)	2.32 ± 0.79 (n = 6)	> 0.05
Sciatic nerve proximal	0.50 ± 0.25 (n = 9)	2.33 ± 1.34 (n = 8)	> 0.05	0.45 ± 0.11 (n = 6)	2.95 ± 1.29 (n = 6)	0.0326
Sciatic nerve distal	0.06 ± 0.01 (n = 10)	1.32 ± 0.91 (n = 7)	> 0.05	0.31 ± 0.08 (n = 6)	0.34 ± 0.15 (n = 6)	> 0.05
Spinal cord	0.75 ± 0.22 (n = 7)	2.32 ± 0.86 (n = 7)	> 0.05	ND	ND	

Following intravenous injection of vectors driving *Egfp* expression in WT mice, VGCNs showed a widespread biodistribution of both vectors in PNS and CNS tissues, with mostly higher VGCNs obtained with AAVrh10 compared to AAV9 but with statistical differences between them only in proximal and distal part of the sciatic nerve (*p* < 0.0001 and *p* = 0.0024 respectively; Fig. 1C; Table 2). However, biodistribution was relatively low in the CNS including brain (0.09 ± 0.02 for AAV9 and 0.25 ± 0.06 for AAVrh10) and spinal cord (0.13 ± 0.02 for AAV9 and 1.0 ± 0.51 for AAVrh10) and much higher in distal more than proximal PNS tissues, showing the reverse gradient of biodistribution compared to the intrathecal injection results. Again, no statistical difference was found between most tissues except in the case of AAVrh10 where there was a difference between the distal sciatic nerve and the trigeminal nerve confirming the gradient from the site near the injection to the most distal site. Thus, VGCNs ranged for AAV9 from 0.46 ± 0.25 in lumbar roots to 0.71 ± 0.24 in distal sciatic nerve and for AAVrh10 from 1.4 ± 0.45 to 2.29 ± 0.67 respectively.

**Table 2:** Vector genome copy numbers (VGCN) in different PNS and CNS tissues of WT mice injected intravenously with AAV9-Mpz.*Egfp* or AAVrh10-Mpz.*Egfp* and of *Gjb1*-null mice injected intravenously with AAV9-Mpz.*GJB1* or AAVrh10-Mpz.*GJB1.* The values represent mean ± SEM.

Tissue	WT mice			Gjb1-null mice		
	AAV9-Mpz.*Egfp*	AAVrh10-Mpz.*Egfp*	*P* value	AAV9-Mpz.GJB1	AAVrh10-Mpz.*GJB1*	*P* value
Lumbar roots	0.46 ± 0.25 (n = 7)	1.40 ± 0.45 (n = 5)	> 0.05	0.21 ± 0.01 (n = 6)	0.09 ± 0.01 (n = 6)	> 0.05
Sciatic nerve proximal	0.14 ± 0.04 (n = 9)	2.00 ± 0.72 (n = 5)	< 0.0001	1.24 ± 0.89 (n = 6)	0.93 ± 0.31 (n = 6)	> 0.05
Sciatic nerve distal	0.71 ± 0.24 (n = 6)	2.29 ± 6.40 (n = 5)	0.0024	1.17 ± 0.47 (n = 6)	2.57 ± 0.64 (n = 6)	0.0335
Spinal cord	0.13 ± 0.02 (n = 7)	1.00 ± 0.51 (n = 3)	> 0.05	0.21 ± 0.07 (n = 6)	0.24 ± 0.16 (n = 6)	> 0.05
Brain	0.09 ± 0.02 (n = 7)	0.25 ± 0.06 (n = 3)	> 0.05	0.13 ± 0.04 (n = 6)	0.03 ± 0.01 (n = 6)	> 0.05
Quadriceps	0.97 ± 0.17 (n = 7)	1.41 ± 0.14 (n = 3)	> 0.05	0.59 ± 0.13 (n = 6)	0.28 ± 0.09 (n = 6)	> 0.05
Trigeminal	0.10 ± 0.01 (n = 7)	0.33 ± 0.11 (n = 5)	> 0.05	0.43 ± 0.21 (n = 6)	0.15 ± 0.04 (n = 6)	> 0.05

Likewise, following intravenous injection of the vectors expressing *GJB1* in *Gjb1*-null mice (Fig. 1D; Table 2), VGCNs ranged for AAV9 from 0.21 ± 0.01 in lumbar roots to 1.17 ± 0.47 in distal nerves and for AAVrh10 from 0.09 ± 0.01 to 2.57 ± 0.64, and were significantly higher only in the distal part of the nerve in the case of AAVrh10, consistent with higher biodistribution in the periphery. However, there was no statistically significant difference between the two vectors besides the distal parts of the nerve (*p* = 0.0335). In the CNS, relatively low VGCNs were obtained with both vectors, ranging from 0.13 ± 0.04 in the brain to 0.21 ± 0.07 in the spinal cord for AAV9, and from 0.03 ± 0.006 to 0.24 ± 0.16 from AAVrh10, respectively. In the trigeminal nerve we obtained VGCNs that were in the same range as in lumbar spinal roots ranging from 0.43 ± 0.21 to 0.1 ± 0.01 for AAV9 and from 0.15 ± 0.04 to 0.33 ± 0.11 for AAVrh10. The gradient was more evident with AAVrh10 resulting in higher VGCNs in the distal compared to the proximal sciatic nerve (*p* = 0.018), the lumbar roots (*p* < 0.0001), and even more the brain (*p* < 0.0001) and trigeminal nerve (*p* < 0.0001). Finally, evaluation of the quadriceps muscle showed strong biodistribution with all intravenously injected vectors and without significant difference between them. Thus, overall, it appears that intravenous injection of AAV9 and AAVrh10 vectors efficiently targets the PNS and especially distal nerves, compared to intrathecal delivery, with a trend for higher biodistribution of AAVrh10. By comparison, the CNS and spinal roots are less efficiently targeted compared to the intrathecal approach.

### EGFP expression following intrathecal and intravenous injection of AAV9-*Mpz*.*Egfp* and AAVrh10-*Mpz*.*Egfp*

**Figure 2:**
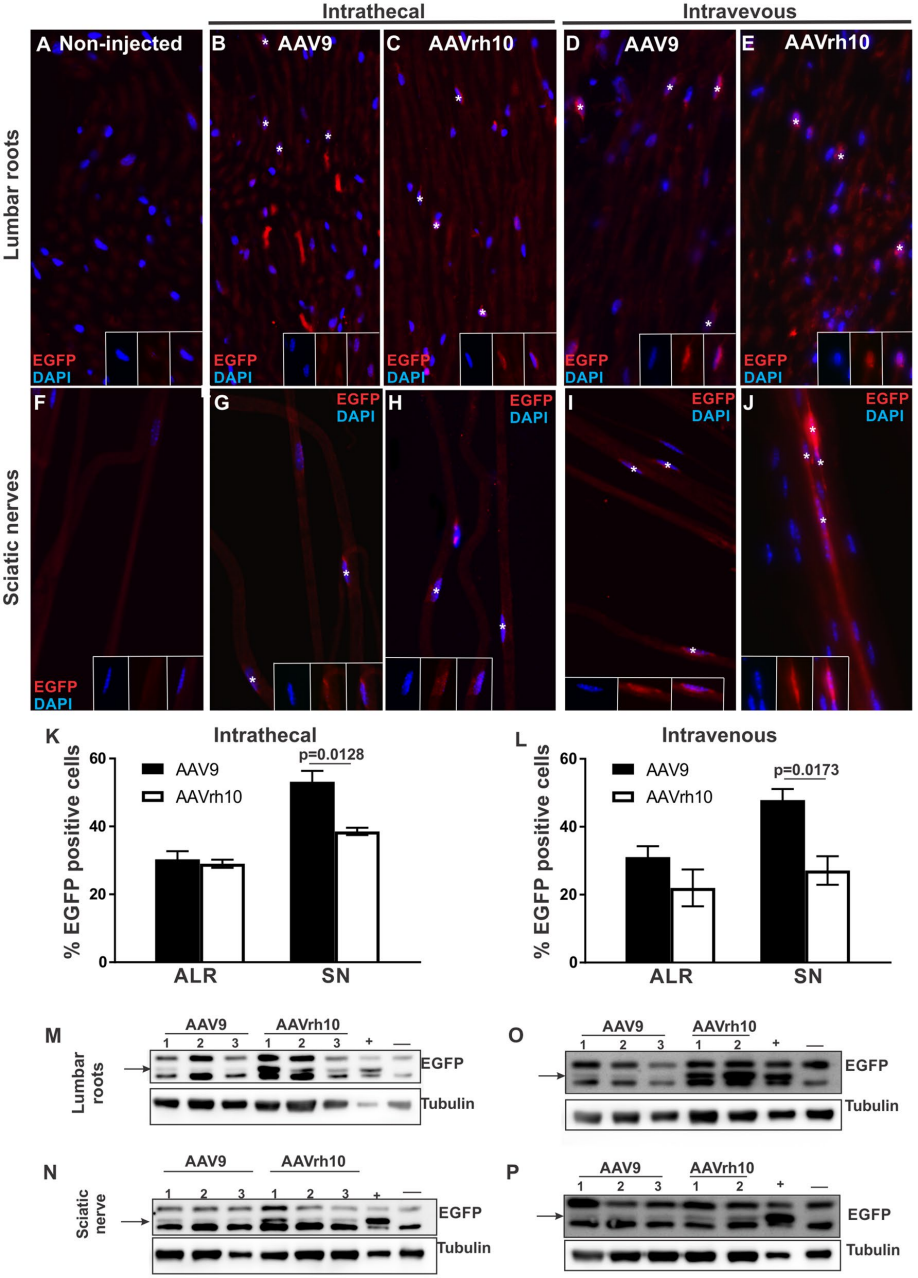
EGFP expression following intravenous or intrathecal injection of AAV9-Mpz.*Egfp* or AAVrh10-Mpz.*Egfp* vectors. Images of anterior lumbar root sections immunostained for EGFP following intrathecal (**B–C**) and intravenous (**D–E**) injection in WT mice show EGFP localization in the perinuclear area (asterisks) of AAV9 (**B, D**) and AAVrh10 (**C, E**) injected mice but not in non-injected mice examined as negative control (**A**). EGFP was also detected in sciatic nerve teased fibers (asterisks) of AAV9 (**G, I**) and AAVrh10 (**H, J**) injected mice but not in the fibers of non-injected mice (**F**). Expression rates after intrathecal (**K**) and intravenous (**L**) delivery of AAV9 and AAVrh10 vectors were similar in anterior lumbar roots (ALR) but higher for AAV9 in the sciatic nerves (SN; *p* = 0.0128 for intravenous injection and *p* = 0.0173 for intrathecal injection). Images were obtained using Nis-Elements version 5.21 (https://www.microscope.healthcare.nikon.com/products/software/nis-elements/nis-elements-advanced-research). EGFP was also detected by immunoblot analysis in anterior lumbar roots (**M**, **O**) and sciatic nerves (**N**, **P**) samples of intrathecally (**M**, **N**) as well as intravenously (**O**, **P**) injected mice, similar to the transgenic positive control ( +) but not in a negative control (−) sample. EGFP levels were higher in AAVrh10 tissues in most cases compared to AAV9 injected mice, but did not show consistent differences between the two administration routes. Data are represented as mean ± SEM. Statistical analysis was performed using Student’s t-test.

Following intrathecal vector injection, we assessed EGFP expression in sections of anterior lumbar roots as well as in both teased fibers and sections of sciatic nerves. EGFP was detected in the perinuclear cytoplasm in a subset of Schwann cells in all injected mice (Fig. 2B, C and G, H) but not in a non-injected WT mouse used as negative control (Fig. 2A, F). Quantification of expression rates showed that 30.3 ± 2.39% (n = 3) of all cells in the lumbar roots of AAV9 injected mice and 29 ± 1.2% (n = 3; *p* > 0.05) in AAVrh10 injected mice were EGFP positive, as well as 53.2 ± 3.25% (n = 3) of cells in the sciatic nerves of AAV9 injected mice and 38.5 ± 1.1% (n = 3; *p* = 0.0128) in AAVrh10 injected mice (Fig. 2K). EGFP expression was confirmed also by immunoblot analysis which detected a specific band in lumbar roots and sciatic nerve samples of intrathecally injected mice with both serotypes as in a transgenic positive control, while it was absent from a negative control sample (Fig. 2M–N). Expression levels showed a trend for higher EGFP expression in AAVrh10-injected mice which was significant only in lumbar roots (*p* = 0.0274) (Suppl. Fig. 1). We did not observe any significant differences between tissues in the expression levels of EGFP following intrathecal injection with either of the two vectors.

Following intravenous injection of EGFP expressing AAV9 or AAVrh10 vectors we similarly examined cryo-sections of lumbar roots and sciatic nerves as well as sciatic nerve teased fibers. EGFP was detected in the perinuclear cytoplasm in a subset of Schwann cells in all injected mice (Fig. 2D, E and I, J) while it was absent from non-injected control (Fig. 2A, F). EGFP expression rates reached 31 ± 3.24% (n = 4) in the lumbar roots of AAV9 injected mice and 22 ± 5.43% (n = 3; *p* > 0.05) in AAVrh10 injected mice; and 47.9 ± 3.26% (n = 4) in the sciatic nerves of AAV9 injected mice and 27.1 ± 4.19% (n = 3; *p* = 0.0173) in AAVrh10 injected mice (Fig. 2L). Immunoblot analysis of lumbar root and sciatic nerve lysates of intravenously injected mice confirmed EGFP expression with both serotypes (Fig. 2O–P). As in intrathecally injected groups, expression levels appeared higher in tissues from AAVrh10 compared to AAV9 injected mice, which was statistically significant only in lumbar roots (*p* = 0.0003) (Suppl. Fig. 1). Higher expression levels in lumbar roots compared to sciatic nerves were observed in AAVrh10 injected mice (*p* < 0.0001). Finally, when comparing the two routes of administration for the same vector, AAV9 showed no differences in the tissues examined, whereas AAVrh10 showed higher EGFP expression levels in lumbar roots of intravenously injected mice compared to those injected intrathecally (*p* = 0.0038).

### Cx32 expression following intrathecal and intravenous injection of AAV9-*Mpz*.*GJB1* and AAVrh10-* Mpz*.*GJB1*

**Figure 3:**
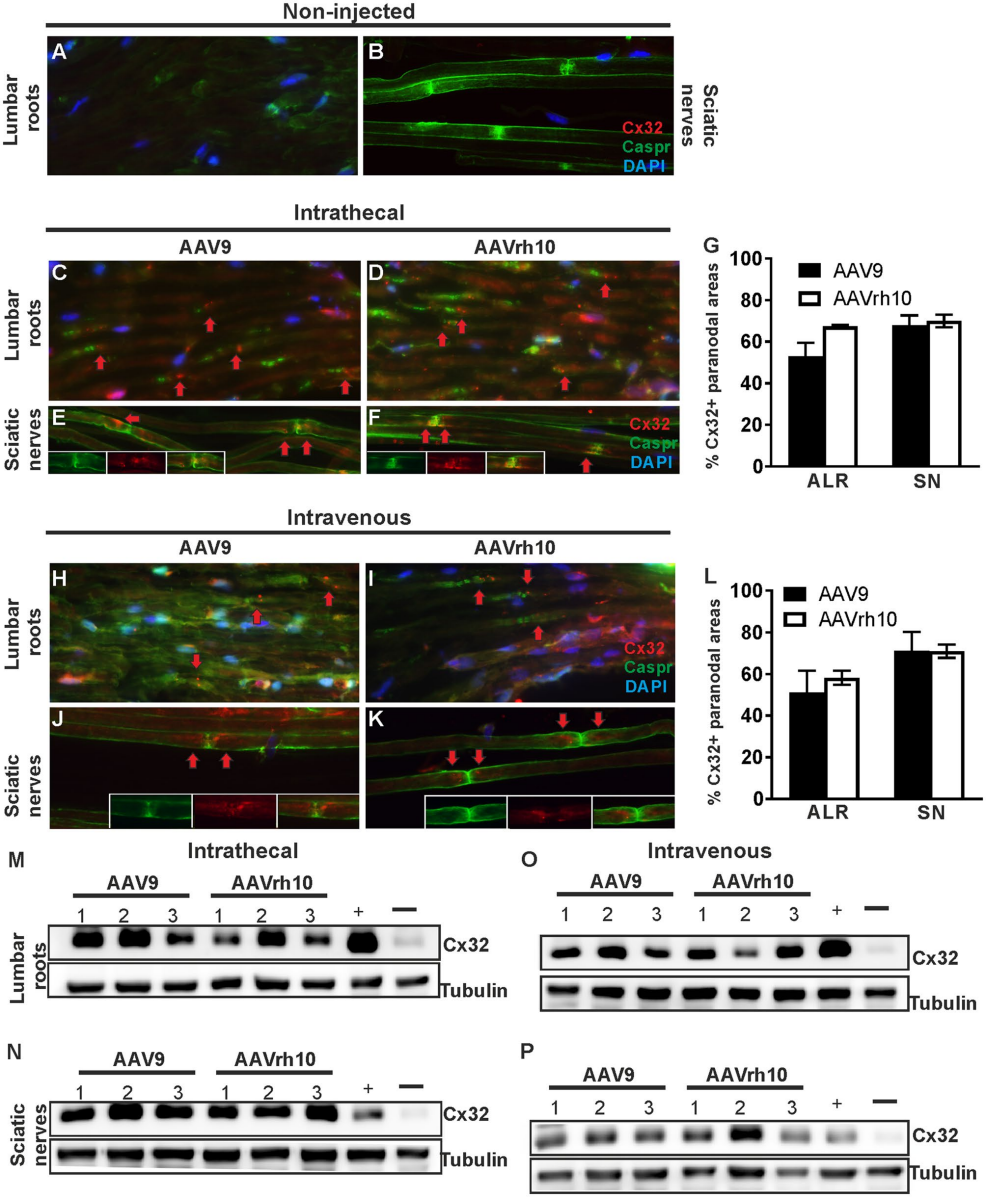
Cx32 expression following intravenous or intrathecal injection of AAV9-Mpz.*GJB1* and AAVrh10-Mpz.*GJB1* in *Gjb1*-null mice. Anterior lumbar root sections of *Gjb1*-null mice were immunostained for Cx32 (red) following intrathecal (**C–D**) or intravenous (**H–I**) vector injection, as indicated. Cx32 is localized at the paranodal non-compact myelin areas (red arrows) of AAV9 (**C**, **H**) and AAVrh10 (**D**, **I**) injected mice as opposed to non-injected mice used as negative control (**A**). Cx32 was also detected in sciatic nerve teased fibers (red arrows) of AAV9 (**E**, **J**) and AAVrh10 (**F**, **K**) injected mice (overview images and magnified nodal areas in separate channels shown in insets) but not in the fibers of non-injected mice (**B**). Expression rates after intrathecal (**G**) or intravenous (**L**) delivery of AAV9 or AAVrh10 were similar in anterior lumbar roots and sciatic nerves across serotypes and administration routes (*p* > 0.05). Images were obtained using Nis-Elements version 5.21 (https://www.microscope.healthcare.nikon.com/products/software/nis-elements/nis-elements-advanced-research). Cx32 was also detected after immunoblot analysis in both anterior lumbar roots (**M**, **O**) and sciatic nerves (**N**, **P**) samples of intrathecally (**M**, **N**) as well as intravenously (**O**, **P**) injected mice, similar to the positive control transgenically expressing the human Cx32 ( +) but not in the non-injected *Gjb1*-null mouse sample used as negative control ( −). Cx32 levels did not show consistent differences between the two vectors and administration routes. Data are represented as mean ± SEM. Statistical analysis was performed using Student’s t-test.

Following intrathecal injection into *Gjb1*-null mice of the *GJB1* expressing vectors we examined Cx32 expression in anterior lumbar roots and sciatic nerves. Cx32 was detected in the paranodal areas corresponding to the non-compact myelin of lumbar root (Fig. 3C–D) and sciatic nerve fibers (Fig. 3E–F), but not in non-injected *Gjb1*-null mice used as negative control (Fig. 3A–B). The percentage of Cx32-positive paranodal areas reached 52.9 ± 6.62% (n = 3) in the lumbar roots of AAV9-injected mice and 67.3 ± 0.71% (n = 3; *p* > 0.05) in AAVrh10-injected mice. Cx32 expression rates in the sciatic nerves reached 67.8 ± 4.82% (n = 3) in AAV9-injected mice and 70.0 ± 3.0% (n = 3; *p* > 0.05) in AAVrh10-injected mice (Fig. 3G). Cx32 expression was confirmed also by immunoblot analysis which detected the specific band in lumbar roots and sciatic nerves of intrathecally injected mice with either serotype, similar to a positive control expressing transgenically human Cx32, while it was absent in the negative control sample from a non-injected *Gjb1*-null mouse (Fig. 3M–N). We observed a trend for higher expression in the AAV9 injected mice compared to the AAVrh10 mice as indicated in Suppl. Fig. 2 but with no statistical difference between the two serotypes. Proportionally higher Cx32 expression levels were observed in sciatic nerves with both serotypes (*p* < 0.0001).

We similarly examined Cx32 expression in cryosections and teased fibers of intravenously injected mice. Virally delivered Cx32 was again detected in the paranodal areas of lumbar roots (Fig. 3H–I) and sciatic nerves (Fig. 3J–K) as expected. Expression rates of Cx32 in intravenously injected mice reached 51.1 ± 10.44% (n = 3) in the lumbar roots of AAV9-injected mice and 58.2 ± 3.32% (n = 3; *p* > 0.05) in roots of AAVrh10-injected mice, as well as 71.1 ± 9.12% (n = 3) in the sciatic nerves of AAV9-injected mice and 71.0 ± 3.2% (n = 3; *p* > 0.05) in AAVrh10-injected mice (Fig. 3L). Similar to the intrathecal injection, Cx32 expression was confirmed also by immunoblot analysis in lumbar roots and sciatic nerve samples of intravenously injected mice with either serotype (Fig. 3O–P). There were no significant differences between the two vectors in the expression levels achieved in all tissues examined (Suppl. Fig. 2). When comparing tissues, we found that sciatic nerves had proportionally higher expression levels compared to lumbar roots with both serotypes (*p* < 0.0001). We further compared expression levels between the two routes of administration but found no significant differences in either lumbar roots or sciatic nerves (*p* > 0.05).

### EGFP expression following intrathecal and intravenous injection of AAV-PHP.S-*CAG*.*Egfp* and AAVrh10-*CAG*.*Egfp*

In order to test the efficacy of another AAV serotype, the AAV-PHP.S, previously reported to efficiently target the PNS^30^, we injected WT mice with AAV-PHP.S*-CAG*.*Egfp* and compared it to the AAVrh10**-***CAG*.*Egfp,* both driving EGFP expression under the ubiquitously active CAG promoter. However, the PHP.S vector could not be produced at the same high titters as the AAVrh10 vector. Intrathecal administration of PHP-S and AAVrh10 vectors at 8.7 × 10^10^ vg in 25 µl, showed high expression in DRG neurons without any differences between the two serotypes (Suppl. Fig. 3A–D). However, sciatic nerves showed high level of transduction with AAVrh10 vector but significantly lower with PHP-S (Suppl. Fig. 3C–F). Percentage of EGFP-positive cells in DRGs reached 73.85 ± 3.6% (n = 3) in the PHP.S-injected mice compared to 83.78 ± 13.5% (n = 3; *p* > 0.05) in the AAVrh10-injected mice. Double staining with the Schwann cell specific marker S100, showed that EGFP signal was detected in more Schwann cells in AAVrh10-injected mice compared to the PHP.S-injected mice (Suppl. Fig. 3E–F). AAV-PHP.S was also delivered by intravenous injection at 1 × 10^12^ vg in 300 µl and expression analysis was conducted. Staining of different tissues for EGFP showed low expression in sciatic nerve, DRGs and higher expression in liver, while there was no expression in the spinal cord (Suppl. Fig. 4A–D). Thus, the PHP-S capsid does not appear to offer any advantage in regard to targeting the peripheral nerves and specifically the myelinating Schwann cells compared to AAVrh10 or AAV9 serotypes.

### Inflammatory responses after intrathecal and intravenous delivery of AAV9 and AAVrh10 vectors

**Figure 4:**
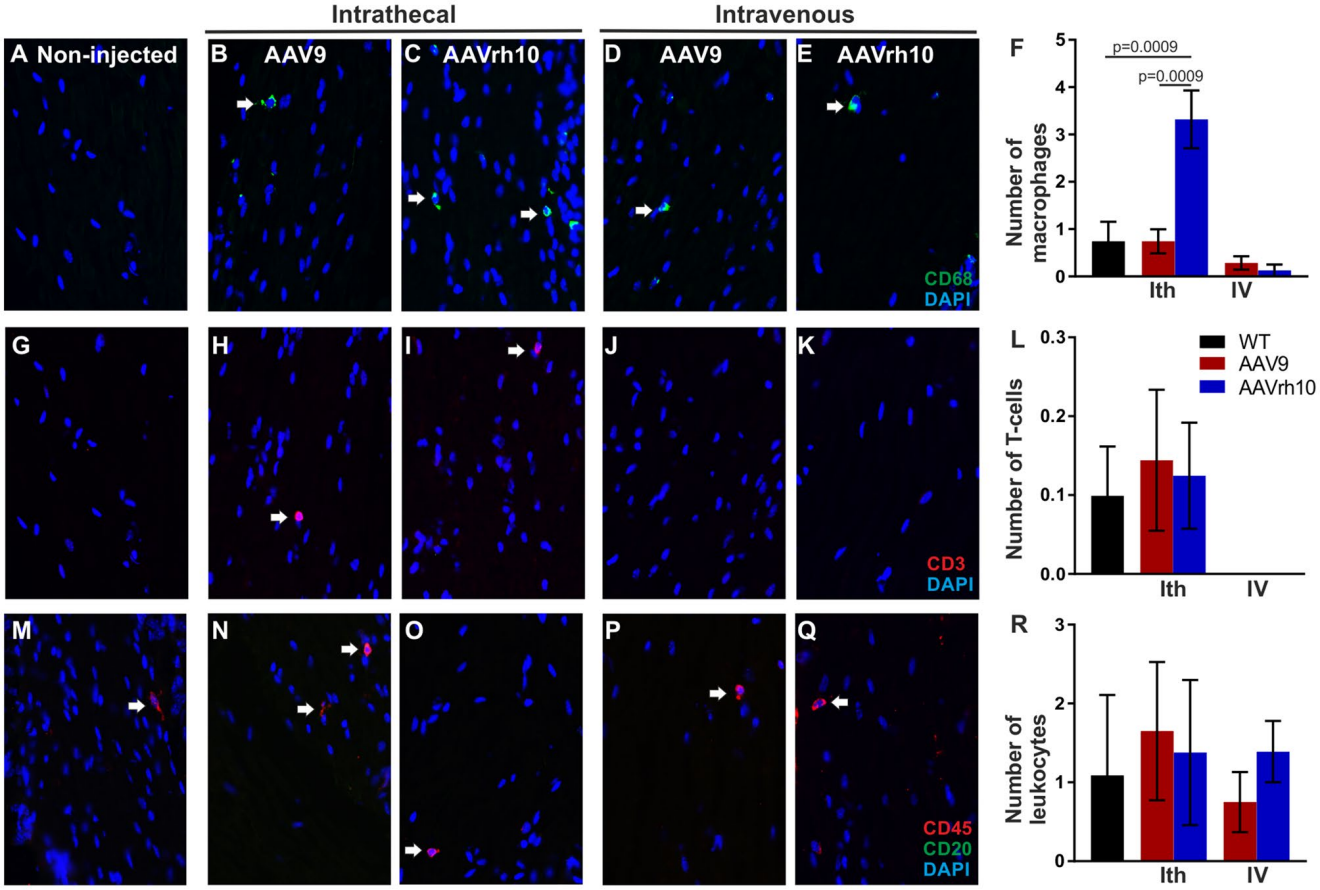
Inflammatory responses in anterior lumbar roots following intravenous and intrathecal injection of AAV9-Mpz.*Egfp* and AAVrh10-Mpz.*Egfp* vectors in WT mice. Macrophages (**A–E**; arrows) were detected in both AAV9 and AAVrh10 injected mice after intrathecal (**B–C**) or intravenous (**D–E**) injection. Images were obtained using Nis-Elements version 5.21 (https://www.microscope.healthcare.nikon.com/products/software/nis-elements/nis-elements-advanced-research). **F**: Quantification showed that macrophage numbers in mice injected intrathecally with AAV9 or intravenously with either AAV9 or AAVrh10 were similar to the non-injected mice, while they were mildly elevated in mice injected intrathecally with AAVrh10. CD3 + T-cells (**G–K**; arrows) were detected only in mice injected intrathecally (**H–I**) but not in intravenously injected animals (**J–K**). Quantification of T-cell numbers showed comparable results between the two serotypes and the non-injected mice (**L**). CD45 + leukocytes (arrows) were also detected in all injected mice with either intrathecal or intravenous approach (**M–Q**). Quantification showed that numbers were similar with both serotypes after either intrathecal or intravenous injection and did not differ significantly compared to non-injected mice (**R**). This was confirmed by two-way ANOVA analysis were there was no significant elevation compared to the wild type or the routes of administration for T cells and leukocytes. Only in macrophages we observed a difference in the numbers in AAVrh10 following intrathecal injection compared to both WT and AAV9 (F(2, 12) = 7.597, *p* = 0.0074) Tukey’s posthoc showed significant difference between the two serotypes compared to the WT (adj. *p* = 0.0009 in both cases). Data are represented as mean ± SEM. Statistical analysis was performed using 2-way ANOVA with Tukey’s post-test.

Inflammatory responses may be a potential limitation to the use of AAV vectors in gene therapy. Therefore, we evaluated by immunostaining lumbar roots, sciatic nerves and liver cryosections for macrophages, T-cells and leukocytes comparing intrathecal and intravenous delivery of AAV9 and AAVrh10 vectors (Fig. 4 and Supp. Fig. 5).

Following intrathecal injection of AAV9-Mpz.*Egfp* and AAVrh10-Mpz.*Egfp* vectors we did not observe any inflammation in the lumbar roots of AAV9-Mpz*.Egfp* injected mice (Fig. 4B) compared to the non-injected WT mice (Fig. 4A). The numbers of macrophages in the AAV9*-*Mpz*.Egfp* injected mice were 0.74 ± 0.25 (n = 3; Fig. 4F) and in non-injected mice 0.74 ± 0.41 (n = 3). In contrast, we observed mild increase in macrophage numbers in AAVrh10*-*Mpz*.Egfp* injected mice (Fig. 4C) reaching 3.32 ± 0.61 (n = 3; *p* = 0.009) compared to the non-injected mice. We further examined the macrophages in sciatic nerves where we observed a small degree of inflammation compared to the non-injected mice. We did not have any macrophages in the non-injected mice while the numbers in the AAV9*-Mpz.Egfp* injected mice reached 0.56 ± 0.56 (n = 3; Supp. Fig. 5) and in AAVrh10*-*Mpz*.Egfp* injected mice 0.24 ± 0.24 (n = 3) both with no statistical difference compared to the WT mice (*p* > 0.05). A small number of CD3 + T-cells were found in lumbar roots after intrathecal injection similar to non-injected WT mice (0.10 ± 0.06; n = 3; *p* > 0.05) (Fig. 4G–I). The CD3 + cell numbers in the AAV9*-*injected mice reached 0.14 ± 0.09 (n = 3; Fig. 4L) while similar were the numbers in the AAVrh10*-*injected mice reaching 0.13 ± 0.07 (n = 3; Fig. 4L). Likewise, in the sciatic nerves of AAV9-injected mice CD3 + cells reached 0.41 ± 0.41 (n = 3; Supp. Fig. 5L and in AAVrh10-injected mice 0.43 ± 0.43 (n = 3) with no statistical difference compared to WT non-injected mice (*p* > 0.05). Finally, CD45 + cell numbers in the injected mice were similar to those of the non-injected mice (1.09 ± 1.09; n = 3; Fig. 4M–O; R). Specifically, CD45 + cells reached in lumbar roots 1.65 ± 0.86 (n = 3) in mice injected with AAV9*-Mpz.Egfp* and 1.38 ± 0.92 (n = 3; *p* > 0.05) with AAVrh10*-*Mpz*.Egfp*. In contrast, we observed CD45 + cells in the sciatic nerves for both serotypes, reaching 3.25 ± 0.63 (n = 3; *p* = 0.0364; Supp. Fig. 5R) for AAV9 and 2.51 ± 0.53 (n = 3) for AAVrh10, with the non-injected mice not presenting any sign of inflammation.

We further evaluated the possibility of inflammation in the liver of mice that were injected intrathecally with either AAV9 or AAVrh10 by staining for macrophages, T-cells and leukocytes and we did not observe any difference to the non-injected mice (Supp. Fig. 7). Inflammatory cell numbers after AAV9*-Mpz.GJB1* and AAVrh10*-Mpz.GJB1* intrathecal injection were similar to the AAV9*-Mpz.Egfp* and AAVrh10*-*Mpz*.Egfp* injected mice as indicated in Suppl. Table 1 and Suppl. Fig. 6.

We further examined potential inflammatory responses following intravenous injection of both AAV9 and AAVrh10 (Fig. 4D–E, J–K, P–Q and Supp. Fig. 5D–E, J–K, P–Q). We counted CD68 + , CD3 + , and CD45 + cells as in tissues of intrathecally injected mice. In lumbar roots numbers of macrophages were similar or even lower compared to the non-injected mice in both serotypes (Fig. 4F; *p* > 0.05). Numbers reached 0.13 ± 0.13 (n = 3) in AAV9-injected mice, 0.29 ± 0.14 (n = 3) in the AAVrh10-injected mice and 0.74 ± 0.41 (n = 3) in the non-injected mice (*p* > 0.05). In the sciatic nerves, AAVrh10-injected mice showed increased CD68 + cells (1.58 ± 0.68; n = 3; *p* = 0.0307) while AAV9-injected mice showed only 0.36 ± 0.19 CD68 + cells (n = 3; Supp. Fig. 5F) but without any statistical difference from the non-injected mice (*p* > 0.05).

We did not observe any CD3 + T-cells in the lumbar roots of the mice injected either with AAV9 or AAVrh10 (Fig. 4L). CD3 + T-cell numbers were lower in the sciatic nerve but again AAVrh10-injected mice showed 0.08 ± 0.08 (n = 3) CD3 + cells compared to no T-cells in AAV9-injected as well as to non-injected mice (Supp. Fig. 5L; *p* > 0.05). Finally, counts of CD45 + cells showed no significant differences compared to the non-injected mice (Fig. 4R), reaching 1.39 ± 0.39 (n = 3) in AAV9-injected, 0.75 ± 0.38 (n = 3) in AAVrh10-injected, and 1.09 ± 1.02 (n = 3; *p* > 0.05) in non-injected mice. In the sciatic nerve we observed some inflammatory responses based on the numbers of CD45 + cells in AAV9-injected (1.72 ± 0.95; n = 3) and AAVrh10-injected (1.52 ± 1.52; n = 3) mice (Supp. Fig. 5R) but without any differences compared to nerves of non-injected mice (*p* > 0.05) in which no CD45 + cells present.

Similar to the intrathecally injected mice, we could not observe any inflammation in the liver of intravenously injected mice. Staining of liver sections for macrophages, T-cells and all leukocytes showed similar negative results to the non-injected mice (Supp. Fig. 7). Finally, results of staining for inflammatory cells in *Gjb1*-null mice after intravenous injection of AAV9*-Mpz.GJB1* and AAVrh10*-Mpz.GJB1* were similar to the AAV9*-Mpz.Egfp* and AAVrh10*-* Mpz*.Egfp* injected mice as indicated in Suppl. Table 1 and Suppl. Fig. 6.

## Discussion

The aim of this study was to optimize the viral vectors and delivery routes for targeting myelinating Schwann cells in the PNS. We selected the AAV9 and AAVrh10 serotypes, which have already been extensively used in both experimental and clinical trials. However, previous studies focused mainly on CNS targeting^9,13,24^, so that the potential of these vectors for targeting the PNS remained unclear. In order to achieve Schwann cell-specific expression, we used the PNS myelin-specific *Mpz* promoter^4,31,32^ and delivered AAV vectors carrying either the reporter gene *Egfp* or the neuropathy associated gene *GJB1* by lumbar intrathecal or by intravenous injection. We demonstrate that both vectors resulted in Schwann cell specific and widespread expression in PNS tissues showing similar biodistribution and gene expression pattern.

In order to develop gene therapies for peripheral demyelinating neuropathies there is a need for targeted gene delivery to the PNS. We have recently shown that AAV9-mediated *GJB1* gene replacement in the PNS can rescue the demyelinating neuropathy in a mouse model of demyelinating neuropathy showing improvement in both morphological and functional properties^4^. However, the vector used in this study had lower titers and with the amount injected reaching 1 × 10^10^ and no comparison was made to any alternative serotype or delivery method to optimize outcomes. For this reason, we wished to expand our approach by testing additional AAV serotypes for their efficacy to transduce Schwann cells as well as to compare the two most translatable routes of administration for targeting the PNS.

This is the first comparison of the two AAV serotypes for targeted gene expression in Schwann cells. The *Mpz* promoter has been previously shown to drive selective expression of gene therapies in Schwann cells using either a lentiviral vector or the AAV9 serotype^4,31^ as well as in studies targeting Schwannomas using the AAV1 serotype^33^. There are also data showing that AAVrh10 results in EGFP expression in feline spinal nerves after intrathecal injection^16^ and in sciatic nerves of diabetic mice injected with AAVrh10-IGF^17^. Although these data suggested that AAVrh10 can transduce tissues of the PNS, this has not been previously directly demonstrated since both EGFP and IGF were driven by a ubiquitous promoter and can be expressed also in axons. This is not the case in our study since expression was driven by the Schwann cell specific *Mpz* promoter, and both vectors showed targeted expression in Schwann cells and not in other cell types.

Furthermore, we provide evidence that both AAV serotypes show adequate biodistribution in relevant PNS tissues as demonstrated by viral genome detection, and can transduce efficiently and with a similar pattern Schwann cells throughout the PNS. Biodistribution analysis showed that both vectors can be found in several other tissues as expected, and especially following intravenous injection, but their expression is limited to peripheral nerve tissues due to the fact that a Schwann cell specific promoter is used. The biodistribution analysis shows an opposite gradient of the VGCNs with the two delivery routes with higher vector numbers in tissues near the injection site as opposed to tissues that are far from the injection site, although in most cases there was no statistical difference between the different tissues. Only AAVrh10 shows a trend for higher accumulation in the distal compared to the more proximal tissues, which is however not reflected in the expression rates. Thus, intrathecal injection results in better and more uniform biodistribution in the desired tissues in addition to the CNS, including the spinal roots and proximal, rather than distal, peripheral nerves, whereas intravenous injection results in better biodistribution to distal more than proximal nerves and spinal roots. Higher vector titers are likely needed to efficiently transduce spinal cord and brain by intravenous delivery, while simultaneous intrathecal and intravenous delivery might provide higher expression also in distal peripheral nerves. This is in accordance with previous studies showing also a gradient pattern in the EGFP expression in the brain following intravenous delivery of AAV9 and AAVrh10 in neonatal mice^34^. Based on our biodistribution data, AAVrh10 appeared to achieve better biodistribution compared to AAV9 in most of the tissues examined. AAVrh10 was also shown to be more efficient in transducing different cell types of the CNS, although it remained close to the efficiency of AAV9^34–36^. Although previous studies suggested that PHP-S may be a suitable vector to target the PNS^30,37^, in our hands it did not provide any advantage compared to AAV9 or AAVrh10 serotypes at least for Schwann cell targeting, although it appears to adequately target DRGs.

Although our biodistribution data show similar distribution of the two vectors, with a trend for statistically insignificant higher VGCNs achieved by AAVrh10, this is not reflected in their respective expression rates in relevant PNS tissues. In most cases, AAVrh10 transgene expression levels remained lower compared to AAV9. This is in contrast to CNS studies showing that AAVrh10 provides higher expression compared to other serotypes^34,35^. This might be either due to the fact that a number of capsids are trapped in different cell types such as ependymal cells, or due to the presence of unproductive viral particles which may result also in CD8 toxicity associated to TLR9 activation^38,39^. In contrast to expression rates, expression levels of EGFP and Cx32 detected by immunoblot analysis tended to be higher with AAVrh10 compared to AAV9 although there was marked variability between samples from different animals. This might be due to the fact that some AAVrh10-transduced cells may produce higher levels of EGFP although the number of the cells transduced was similar with both vectors. Taken together with the expression rates obtained by immunostaining, this may indicate that compared to AAV9, AAVrh10 can transduce fewer Schwann cells but the expression in these cells is higher. Based on these results, AAV9 appears to be more efficient compared to AAVrh10 providing a more balanced expression pattern in target cells and likely a better DNA to expression ratio. These findings may need further validation in other species such as NHPs, in which cell tropism and DNA to expression ratio may be different compared to rodents.

Further to the comparison of the relevant AAV serotypes, in this study we also directly compared the efficacy of lumbar intrathecal and intravenous injection to target the PNS. This is the first time that these two most clinically relevant routes of administration are compared for PNS targeting. In our previous studies so far targeting the PNS, we used either direct intraneural^17,40^ or lumbar intrathecal injection^4,7,31,41^. Our comparison of intrathecal to intravenous injection indicated that despite the reverse gradient of vector biodistribution depending on the injection route as outlined above, expression levels in PNS tissues were similar. This is in accordance with the study of Chen et al. (2020) showing that intrathecal and intravenous delivery result in similar rescue of aspartyglucosaminuria when treated with the AAV9 using similar doses to those used in our study^27^. Since both administration routes are clinically translatable and provide similar efficacy and expression rates for gene delivery, while a fivefold lower volume is needed for intrathecal delivery, the intrathecal approach may be preferable for gene therapy targeting the PNS. Although promising, intrathecal injection has to be further tested in non-human primates for its efficacy and the potential for scale-up. Data concerning the efficacy of intrathecal delivery in non-human primates are different depending on vectors and site of injection used. Ramsingh et al. (2018) showed efficient transduction using AAV9^42^, while Hinderer et al. (2020) showed that intrathecal injection of AAVrh78 failed to transduce the CNS in older animals using large injection volumes but was efficient in younger animals. In contrast, intra-cisterna magna injection proved to be efficient in both young and older animals at lower volumes^29^. Further, a suboccipital administration into the CSF (intrathecal) of infant monkeys of AAV9 resulted in sustained expression up to 4 years showing that intrathecal injection is an efficient and safe way of delivery^43^. In this study we used 20 μl which is injected gradually in the CSF with a rate of 5 μl/min and although the volume of the mouse CSF is 35 μl^44^ there is no disruption of the barrier as Hinderer et al. (2020) suggest^29^.

Since safety and immunological reaction to vector injections for gene therapy are an important consideration before moving to clinical translation^18,25^, we further analysed the possible inflammatory and toxic responses caused by the two routes of administration using the two AAV vectors. Our data show that overall numbers of immune cells in relevant tissues are similar to the non-injected animals for both serotypes and routes of administration used, although both the intrathecal and intravenous delivery of AAvrh10 increases the numbers of macrophages in tissues near the site of delivery and in particular in lumbar roots following intrathecal delivery and in sciatic nerve following intravenous delivery. Despite the high titers of the vector injected and the high VGCNs in the liver (data not shown) no toxic effects were observed in the liver with either vector or administration route. This indicates that both intrathecal and intravenous injection of either of the two serotypes is overall safe, whereby AAV9 appears to cause lower inflammatory responses. No or mild toxicity was also observed by Hordeaux et al.^25^ in a toxicology study after intra-cisterna magna injection in non-human primates, while Gushchina et al. (2021) showed no toxicity even after high doses of AAV9.^45^. Although these are encouraging data supporting the safety of AAV vectors, there are also data showing that both vectors can have toxic effects after intravenous injection in both non-human primates^19^ and mice^18^. Importantly, there are reports of hepatotoxic effects also in humans following delivery of AAVs, raising more concerns about their safety^46,47^. Thus, further studies of hepatotoxicity are needed to ensure patient safety in future clinical applications.

Intrathecal delivery might be the most applicable solution for gene therapy using AAV vectors since data show that this type of injection can overcome the immune response attributed to pre-existing AAV antibodies. Pre-existing AAV antibodies can modulate the safety and efficacy of the vectors used for gene therapy by blocking vector transduction or redirecting distribution of AAV vectors to target other organs than the ones desired^48^. Gray et al. (2013) also showed that intrathecal injection in non-human primates can overcome the issue of pre-existing antibodies^9^. These data show that our approach for intrathecal delivery is the safest and easiest way to use the AAVs for gene therapy.

In conclusion, this study provides compelling evidence of the efficacy of both AAV9 and AAVrh10 to target the PNS and transduce Schwann cells for gene therapy, with AAV9 appearing to be more efficient compared to AAVrh10. We further show that two clinically translatable routes of administration can be used for PNS targeting with the lumbar intrathecal delivery being more efficient since lower volumes are required to achieve similar expression rates throughout the PNS. Our data need to be further validated and especially confirmed in non-human primates in order to get more clinically translatable results for the efficacy and safety of both serotype and route of administration to treat inherited demyelinating neuropathies.

## Materials and methods

### Cloning and production of AAV vectors

We generated novel constructs for AAV vector gene delivery designed to provide Schwann cell-specific expression of either the reporter gene EGFP (pAAV-*Mpz.Egfp,* mock vector; Fig. 1A) or of Cx32 (pAAV-*Mpz.GJB1,* full vector; Fig. 2A) as recently described^4^, both under the 1.2 kB *Mpz* promoter shown to drive expression specifically in Schwann cells^31,32^. Briefly, these vectors have been cloned using as starting plasmid the AAV construct pAM/Mbp-EGFP-WPRE-bGH38*,* containing the woodchuck hepatitis virus post-transcriptional regulatory element (WPRE) and the bovine growth hormone polyadenylation sequence (bGHpA) flanked by AAV2 inverted terminal repeats. Cloning of chicken ß-actin promoter with CMV enhancer and WPRE sequences regulating the sequence of GFP were previously described^17^.

Recombinant AAV9, AAVrh10 and PHP.S (rAAV) vectors were produced as already described in previous studies^18^ at the Viral Production Unit of UAB-VHIR (www.viralvector.eu) by triple transfection into HEK293-AAV cells of the expression plasmids, Rep2Cap9 or Rep2Caprh10 (provided by J.M. Wilson, University of Pennsylvania, Philadelphia, USA) or PHP.S plasmids (provided by V. Gradinaru, California Institute of Technology, Pasadena, CA, USA) containing AAV genes and pXX6 plasmid containing adenoviral genes^49^ needed as helper virus. AAV particles were purified by iodixanol gradient after benzonase treatment. Titration was evaluated by picogreen (Invitrogen)^50^ quantification and calculated as viral genomes per milliliter (vg/ml).

### Experimental animals

We used 2-month-old WT C57BL/6 mice (n = 6–10 per vector and injection method) for pAAV-*Mpz.Egfp* injection and EGFP expression analysis, as well as 2-month-old *Gjb1-*null/Cx32 KO (C57BL/6_129) mice (n = 6 per vector and injection method) for pAAV-*Mpz.GJB1* injection and Cx32 expression analysis, all weighing 20–25 g. *Gjb1-*null/Cx32 KO mice were obtained from the European Mouse Mutant Archive, originally generated by Prof. Klaus Willecke (University of Bonn). All experimental procedures in this study were conducted in accordance with animal care protocols approved by the Cyprus Government’s Chief Veterinary Officer legally responsible in Cyprus for approving animal studies according to EU regulations with an ethics committee (project license CY/EXP/PR.L3/2017) according to national law, which is harmonized with EU guidelines (EC Directive 86/609/EEC). ARRIVE guidelines have been followed in this study.

### Intrathecal and intravenous vector delivery

We delivered the AAV vectors by lumbar intrathecal as previously described^31,51^ or by intravenous injection. For intrathecal injection, a small skin incision was made along the lower lumbar spine level of anesthetized mice to visualize the spine and the AAV vector was delivered into the L5-L6 intervertebral space. A 50-μL Hamilton syringe (Hamilton, Giarmata, Romania) connected to a 26- or 30-gauge needle was used to inject 20 µL of AAV stock containing an estimated 2 × 10^11^ vector genomes (vg), at a maximum rate of 5 µL/min. A flick of the tail was considered indicative of successful intrathecal administration. For intravenous injection an insulin syringe was inserted in the tail vein and 100 µL of the vector were injected containing an estimated 1 × 10^12^ vg.

### Vector genome copy number (VGCN) determination

VGCN was determined as already described in previous study^4^. Briefly, genomic DNA was extracted from different PNS and CNS tissues (i.e., lumbar roots, proximal, middle and distal sciatic nerve, femoral nerve, brain, liver, trigeminal and spinal cord) of mice 6 weeks after intrathecal and intravenous vector delivery using the MagPurix Tissue DNA Extraction Kit (Zinexts Life Science. The extracted DNA was analyzed for yield and purity using a Nanodrop 1000 spectrophotometer. Approximately 20 ng of DNA was used as template for two real-time PCR assays on an Applied Biosystems 7500 Real-Time PCR System involving 45 cycles of 15 s at 95 °C and 60 s at 60 °C. TFRC-specific primers/probe targeting the mouse genome and WPRE-specific primers/probe, which is contained in the transgene, were used. Standard curves were created by serial dilution of quantified mouse genomic DNA, as well as quantified plasmid DNA containing the transgene cassette. The average VCN per cell was calculated as the total VCN divided by the total cell number.

### Immunofluorescence staining

Immunostaining was performed according to previous published protocols^4,31,40,41,52^. For immunostaining, mice were anesthetized with avertin according to institutionally approved protocols, and then transcardially perfused with normal saline followed by fresh 4% paraformaldehyde in 0.1 M PB buffer. The lumbar-sacral spinal cords with spinal roots attached, as well as the bilateral sciatic and femoral motor nerves were dissected. Spinal cord along with the lumbar roots and sciatic and femoral nerves were post-fixed for 2 h and 30 min respectively. Spinal cords with roots attached and parts of sciatic nerves were frozen for cryosections, while parts of sciatic and femoral nerves were isolated and teased into fibers under a stereoscope. Teased fibers or sections were permeabilized in cold acetone and incubated at RT with a blocking solution of 5% BSA (Sigma-Aldrich, Munich, Germany) containing 0.5% Triton-X (Sigma-Aldrich, Munich, Germany) for 1 h. Primary antibodies used were: mouse monoclonal antibody against contactin-associated protein (Caspr, 1:50; gift of Dr Elior Peles, Weizmann Institute of Science), rabbit antisera against EGFP (1:1,000; Invitrogen, USA), Caspr2 (1:200, Alomone Labs, Israel), Schwann cell marker S100 (1:1000; DakoCytomation, Glostrup, Denmark), and Cx32 (1:50; Invitrogen, USA) all diluted in blocking solution and incubated overnight at 4 °C. Slides were then washed in PBS and incubated with mouse cross-affinity fluorescein-conjugated (1:1000; Invitrogen, USA) and rabbit cross-affinity purified rhodamine-conjugated (1:500; Jackson ImmunoResearch, USA) secondary antibodies for 1 h at RT. Cell nuclei were visualized with DAPI (1 µg/ml; Sigma, Munich, Germany). Slides were mounted with fluorescent mounting medium and images photographed under a fluorescence microscope with a digital camera using a fluorescence microscope (Nikon Eclipse N*ἱ*; Tokyo, Japan) with digital camera (DS-Qi2) using NIS-Elements AR 5.21 software (https://www.microscope.healthcare.nikon.com/products/software/nis-elements/nis-elements-advanced-research).

EGFP positive cells and total number of cells were quantified and EGFP positivity rates were expressed as a percentage of the total cell numbers. For quantification of Cx32 expression, Cx32-positive paranodal areas were quantified and expressed as percentage of total paranodal areas in each section or teased fiber preparation. For DRG neuron counting, one every five 10-µm thick sections was selected, and DRG neurons were identified as large, round cell bodies with large round nuclei, surrounded by satellite cells with small, elongated nuclei. At least five sections for each DRG were counted. EGFP was detected by direct fluorescence, without antibody enhancement.

We further studied inflammatory responses following either intrathecal or intravenous injection using cryosections of lumbar roots, sciatic nerves and liver. Sections were stained with rat CD68 (1:50: Serotec), a macrophages marker, rat CD45 (1:100; Abcam), a common leukocytes marker, rabbit antisera CD3 (1:100; Abcam), a T-cells marker, and finally, goat CD20 (1:100, Santa Cruz), a B-cells marker all diluted in blocking solution and incubated overnight at 4 °C. Slides were then washed in PBS and incubated with rat cross-affinity rhodamine-conjugated (1:2000; Invitrogen, USA), goat cross-affinity fluorescein-conjugated (1:700; Abcam) and rabbit cross-affinity purified rhodamine-conjugated (1:500; Jackson ImmunoResearch, USA) secondary antibodies for 1 h at RT. Cell nuclei were visualized with DAPI (1 µg/ml; Sigma, Munich, Germany) or Hoescht solution (Sigma). Slides were mounted with fluorescent mounting medium and images photographed under a fluorescence microscope with a digital camera using a fluorescence microscope (Nikon Eclipse N*ἱ*; Tokyo, Japan) with digital camera (DS-Qi2) using NIS-Elements software. CD positive cells and total number of cells were quantified and CD positive cells rates were expressed as a percentage of the total cell numbers.

### Immunoblot analysis

Fresh sciatic and femoral nerves and lumbar spinal roots were collected at 6 weeks post-injection and lysed in ice-cold RIPA buffer (10 mM sodium phosphate, pH 7.0, 150 mM NaCl, 2 mM EDTA, 50 mM sodium fluoride, 1% Nonidet P-40, 1% sodium deoxycholate, and 0.1% SDS) containing a mixture of protease inhibitors (Roche). Proteins (150 μg) from the lysates were fractionated by 12% SDS/PAGE and then transferred to a Hybond-C Extra membrane (GE Healthcare Life Sciences) using a semidry transfer unit. Nonspecific sites on the membrane were blocked with 5% non-fat milk in PBS with Tween 20 (PBST) for 1 h at room temperature. Immunoblots were incubated with rabbit antisera against EGFP (1:1,000; Abcam) or Cx32 (clone 918, 1:3,000)^53^ and mouse β-tubulin (1:4,000; Developmental Studies Hybridoma Bank) at 4 °C overnight. After washing, the immunoblots were incubated with an anti-mouse or anti-rabbit HRP-conjugated secondary antiserum (Jackson ImmunoResearch, diluted 1:3,000) in 5% milk–PBST for 1 h. The bound antibody was visualized by an enhanced chemiluminescence system (GE Healthcare Life Sciences).

### Statistical analysis

The percentages of EGFP-positive Schwann cells or Cx32-expressing paranodal myelin areas in immunostained spinal roots and sciatic nerves of WT and *Gjb1*-null mice injected with the mock or full vector, respectively, were compared with Student’s t-test using GraphPad Instat3 software (GraphPad, USA). 2-way ANOVA with Tukey’s post-test was used for the comparison of VGCNs of AAV9 vs. AAVrh10, for the comparison of EGFP and Cx32 expression in the immunoblot analysis and for comparison of inflammatory responses following intrathecal injection of both vectors and both delivery methods using Prism 7 (GraphPad, USA).

## Supplementary Information

Supplementary Information.
