# Supplementary material for: Efficacy of AAV serotypes to target Schwann cells after intrathecal and intravenous delivery
Source: Sci Rep. 2021 Dec 2;11:23358. doi: 10.1038/s41598-021-02694-1 (PMC8640002; doi:10.1038/s41598-021-02694-1)
Supplement: Supplementary file 1 — Supplementary Information. [file 41598_2021_2694_MOESM1_ESM.pdf]

Supplementary Material

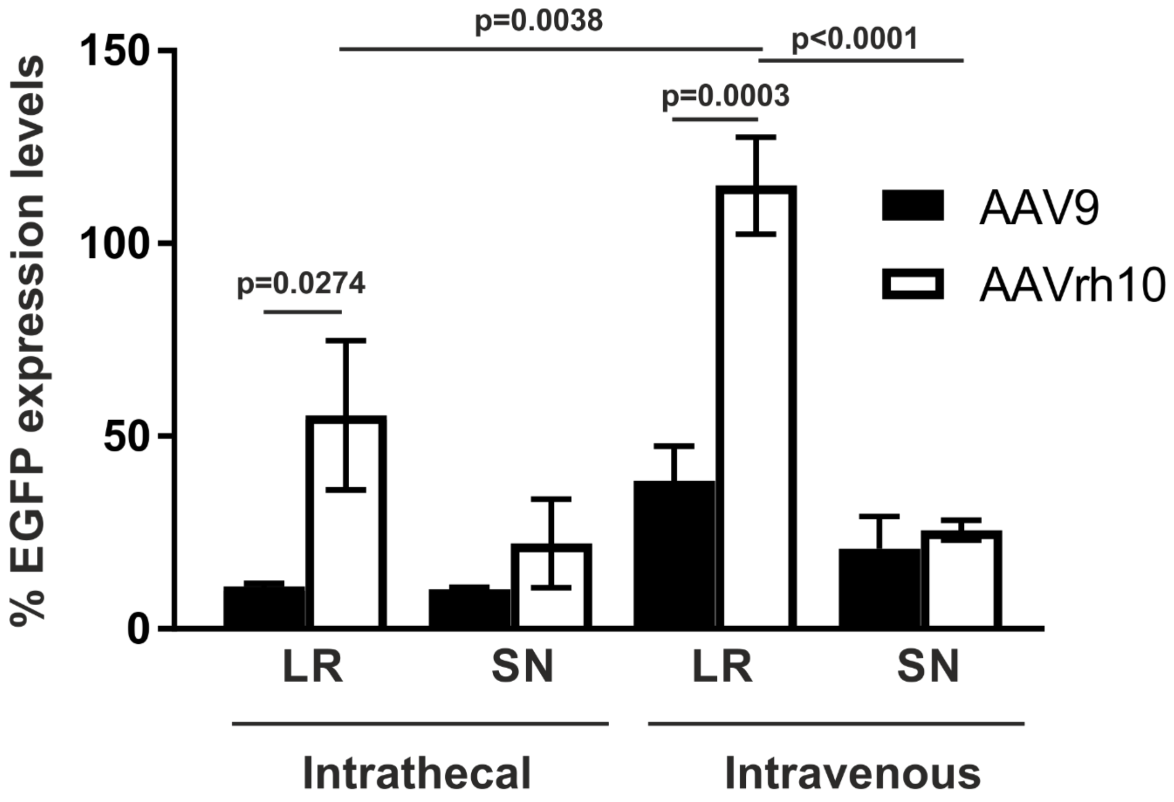

Supplementary Figure 1: EGFP expression levels following intrathecal or intravenous injection of AAV9-Mpz.*Egfp* or AAVrh10-Mpz.*Egfp* vectors. Following intrathecal injection of AAV9-Mpz.*Egfp* or AAVrh10-Mpz.*Egfp* there is a trend for higher EGFP expression levels in AAVrh10-Mpz.*Egfp* injected mice which is statistically significant only in lumbar roots (LR) as revealed by two-way ANOVA ($F(1, 16)=22.97$, $p=0.0002$). Tukey's posthoc confirmed the difference between the two serotypes (adj. $p=0.0274$). Likewise, following intravenous injection, AAVrh10-Mpz.*Egfp* injected mice showed significantly higher EGFP expression levels only in lumbar roots as revealed by two-way ANOVA ($F(1, 16)=22.97$, $p=0.0002$). Similar to the intrathecal injection there was significant difference between the two serotypes revealed by the posthoc analysis (adj. $p=0.0003$). Comparison of the two routes of administration showed increased expression in lumbar roots of intravenously compared to intrathecally injected AAVrh10-Mpz.*Egfp* mice as indicated by two-way

ANOVA analysis ($F(3, 16)=14.33$, $p<0.0001$) This was further confirmed by the Tukey's posthoc test (adj. $p=0.0038$), while there were no differences observed in sciatic nerves (SN) with both delivery routes. When comparing lumbar roots with sciatic nerves there was a trend for higher expression levels in the roots but this was significant only in intravenously AAVrh10-Mpz.*Egfp* injected mice as indicated by two-way ANOVA analysis (adj. $p<0.0001$). Statistical analysis was performed using 2-way ANOVA with Tukey's post-test.

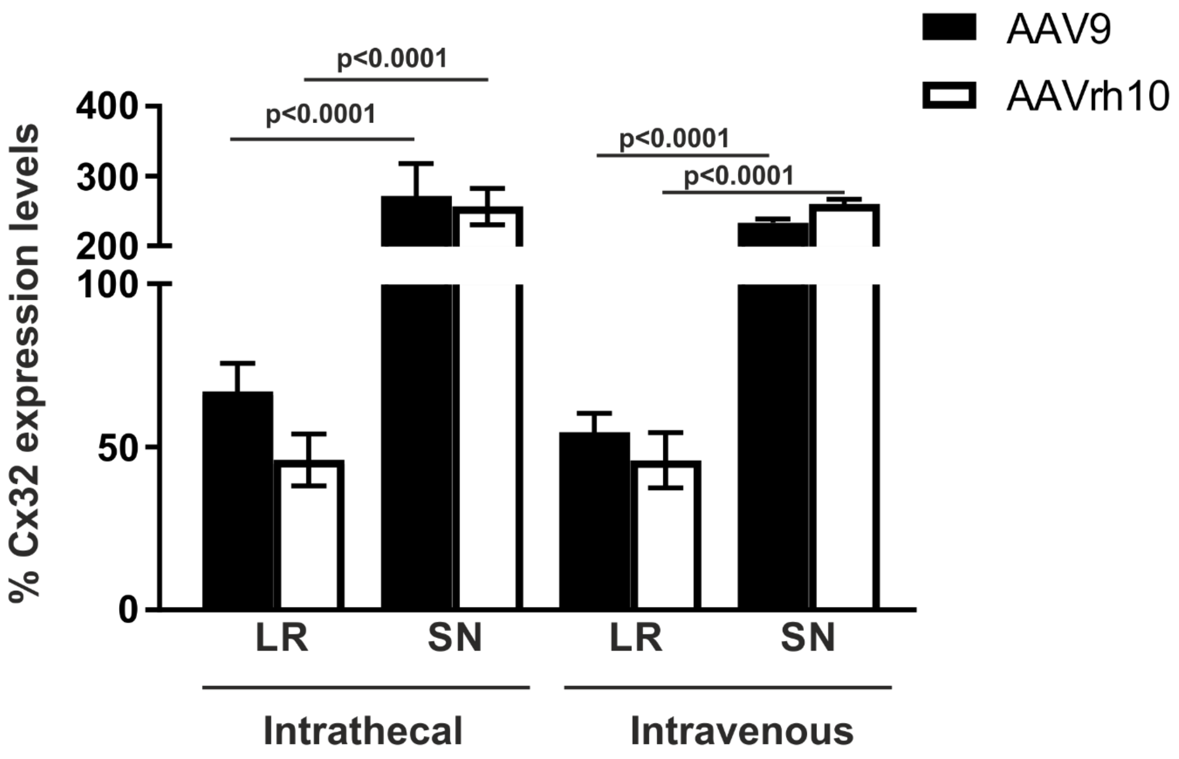

Supplementary Figure 2: Cx32 expression levels following intrathecal or intravenous injection of AAV9-Mpz.*GJB1* and AAVrh10-Mpz.*GJB1* vectors. Following intrathecal injection there is a trend for higher Cx32 expression levels in lumbar roots (LR) of AAV9-Mpz.*GJB1* compared to AAVrh10-Mpz.*GJB1* injected mice but without reaching statistical significance. Likewise, following intravenous injection, AAV9-Mpz.*GJB1* injected mice show a trend for higher Cx32 expression levels in lumbar roots compared to AAVrh10-Mpz.*GJB1* injected mice without statistical significance, while Cx32 expression levels in sciatic nerves showed a trend for increase in AAVrh10-Mpz.*GJB1* injected mice compared to the AAV9-Mpz.*GJB1* injected mice. No differences were observed between the two routes of administration in corresponding tissues or between the serotypes. Significantly increased Cx32 expression levels were observed in sciatic nerves when compared to the lumbar roots with both vectors and with both routes of administration as indicated by two-way ANOVA ($F(3, 16)=70.45$, $p<0.0001$). This was revealed also with the Tukey's posthoc test in all

cases(p<0.0001). Statistical analysis was performed using 2-way ANOVA with Tukey's post-test.

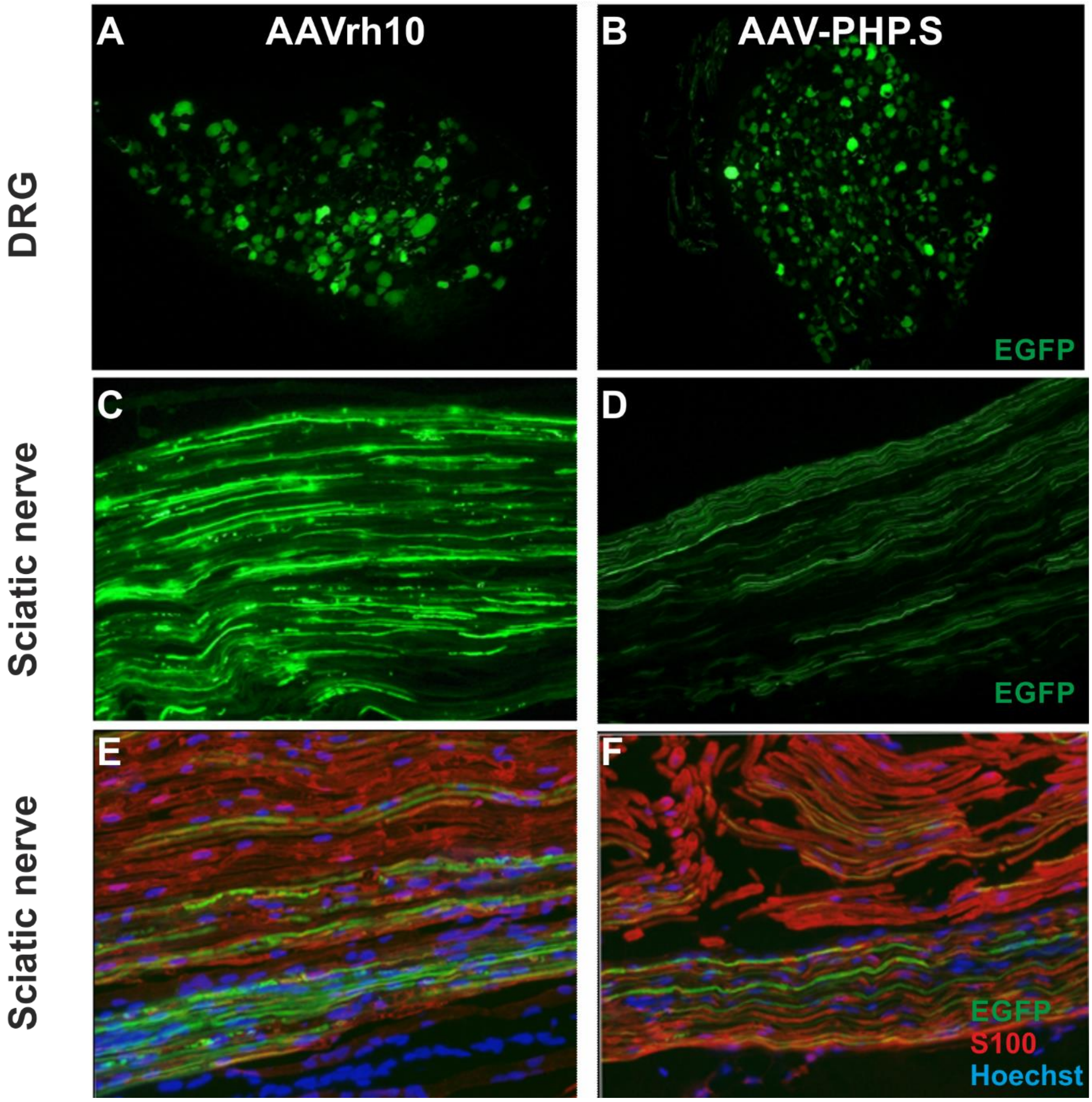

Supplementary Figure 3: EGFP expression following intrathecal injection of AAVrh10-CAG.*Egfp* or AAV-PHP.S-CAG.*Egfp* vectors. A-B: Images of dorsal root ganglia (DRG) sections from intrathecally injected WT mice showing direct EGFP expression (green) in DRG cells with similar efficacy of both vectors. **C-F:** EGFP was also detected in sciatic nerve sections of mice injected with the same vectors with AAVrh10 showing higher EGFP expression levels compared to PHP-S mostly detected in axons (**C-D** single channel and **E-F**

overlay images). EGFP was also detected in Schwann cells in AAVrh10 injected animals (**E**) as indicated by double staining with a Schwann cell marker S100 (red). Nuclei are counterstained with Hoechst (blue).

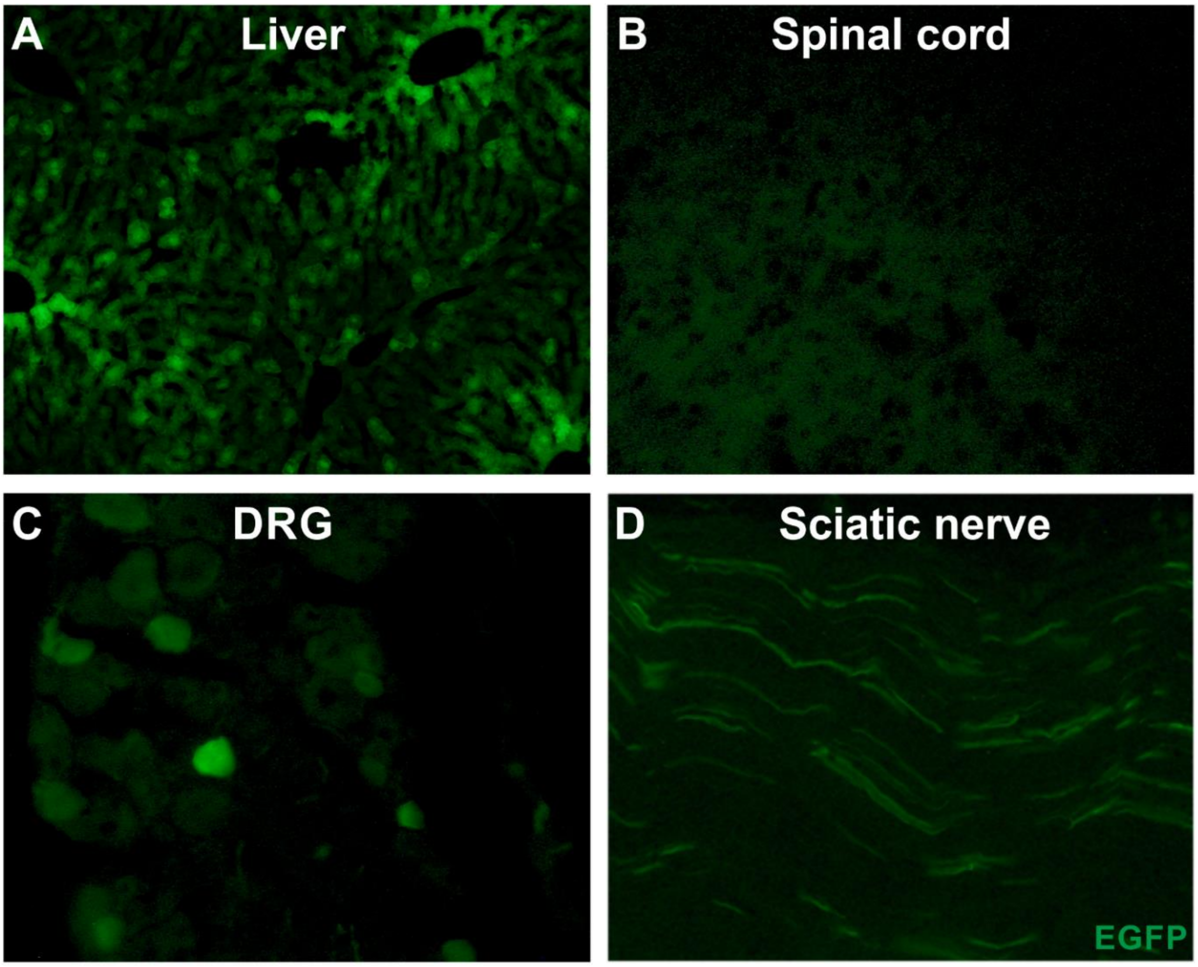

Supplementary Figure 4: EGFP expression following intravenous injection of AAV-PHP.S-CAG.*Egfp* vector. Images of liver (**A**), spinal cord (**B**), DRGs (**C**) and sciatic nerve (**D**) sections following intravenous injection of AAV-PHP.S-CAG.*Egfp* in WT mice show EGFP expression (green) mostly in the liver and lower signal in DRGs and sciatic nerve while no expression was detected in the spinal cord.

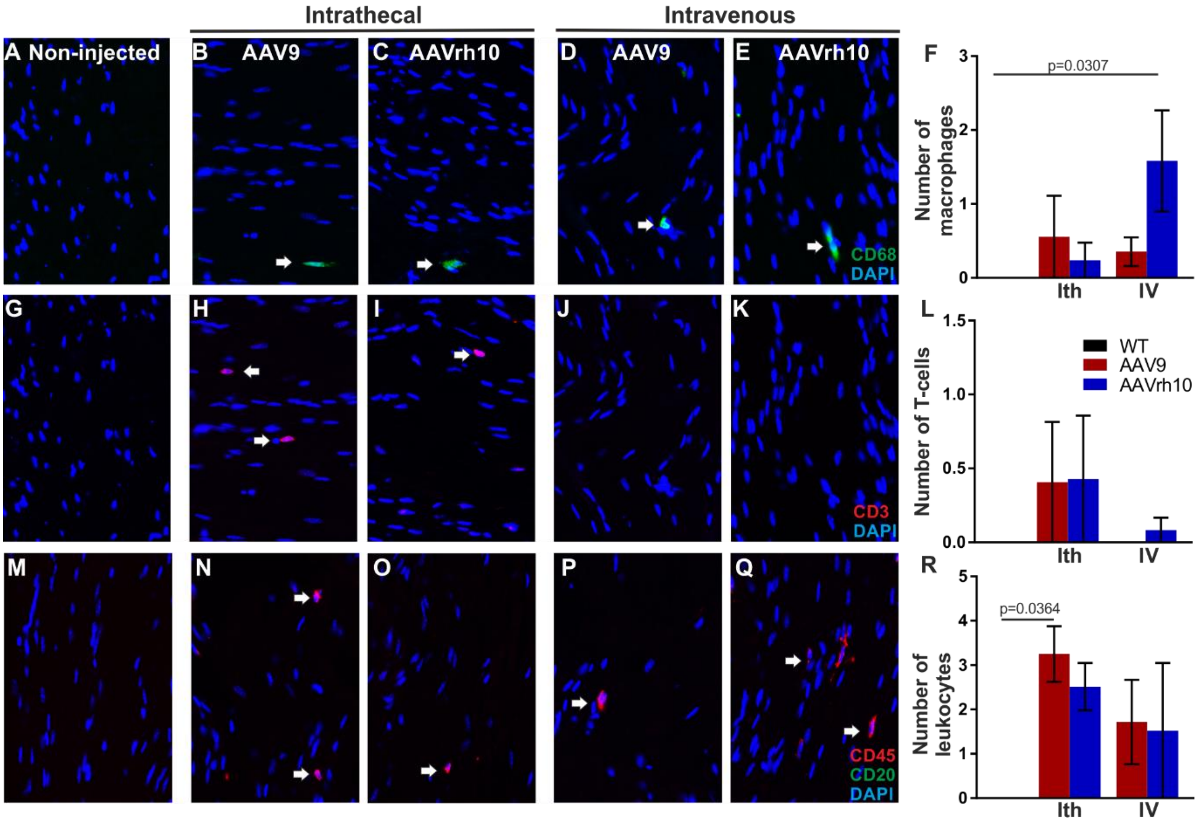

Supplementary Figure 5: Inflammatory responses in sciatic nerves following intrathecal or intravenous injection of AAV9-Mpz.*Egfp* and AAVrh10-Mpz.*Egfp* vectors in WT mice. Macrophages (**A-E**; arrows) were detected in both AAV9 and AAVrh10 injected mice after intrathecal (**B-C**) or intravenous (**D-E**) injection. **F**: Quantification showed that macrophage numbers in mice injected intrathecally with AAV9 or intravenously with either AAV9 or AAVrh10 were similar to the non-injected mice, while they were mildly elevated in mice injected intravenously with AAVrh10. CD3+ T-cells (**G-K**; arrows) were detected mostly in mice injected intrathecally (**H-I**) but not in intravenously injected animals (**J-K**). Quantification of T-cell numbers showed comparable results between the two serotypes (**L**). CD45+ leukocytes (arrows) were also detected in all injected mice with either intrathecal or intravenous approach (**M-Q**). Quantification showed that numbers were similar with both serotypes after either intrathecal or intravenous injection (**R**). Two-way ANOVA revealed differences only when compared to the WT indicating a slight toxicity either after intravenous injection of the AAVrh10 in the numbers of macrophages ($F(2, 12)=2.866$, $p=0.0961$) or

intrathecal injection of AAV9 in the numbers of leukocytes (F(2, 12)=5.352, p=0.0218) Macrophages were increased in AAVrh10 (adj. p=0.0307) and leukocytes were increased in AAV9 (adj. p=0.0364) injected mice compared to WT as Tukey's posthoc showed. There were no differences between the two routes of administration or between the two serotypes as indicated by two-way ANOVA. Statistical analysis was performed using 2-way ANOVA with Tukey's post-test.

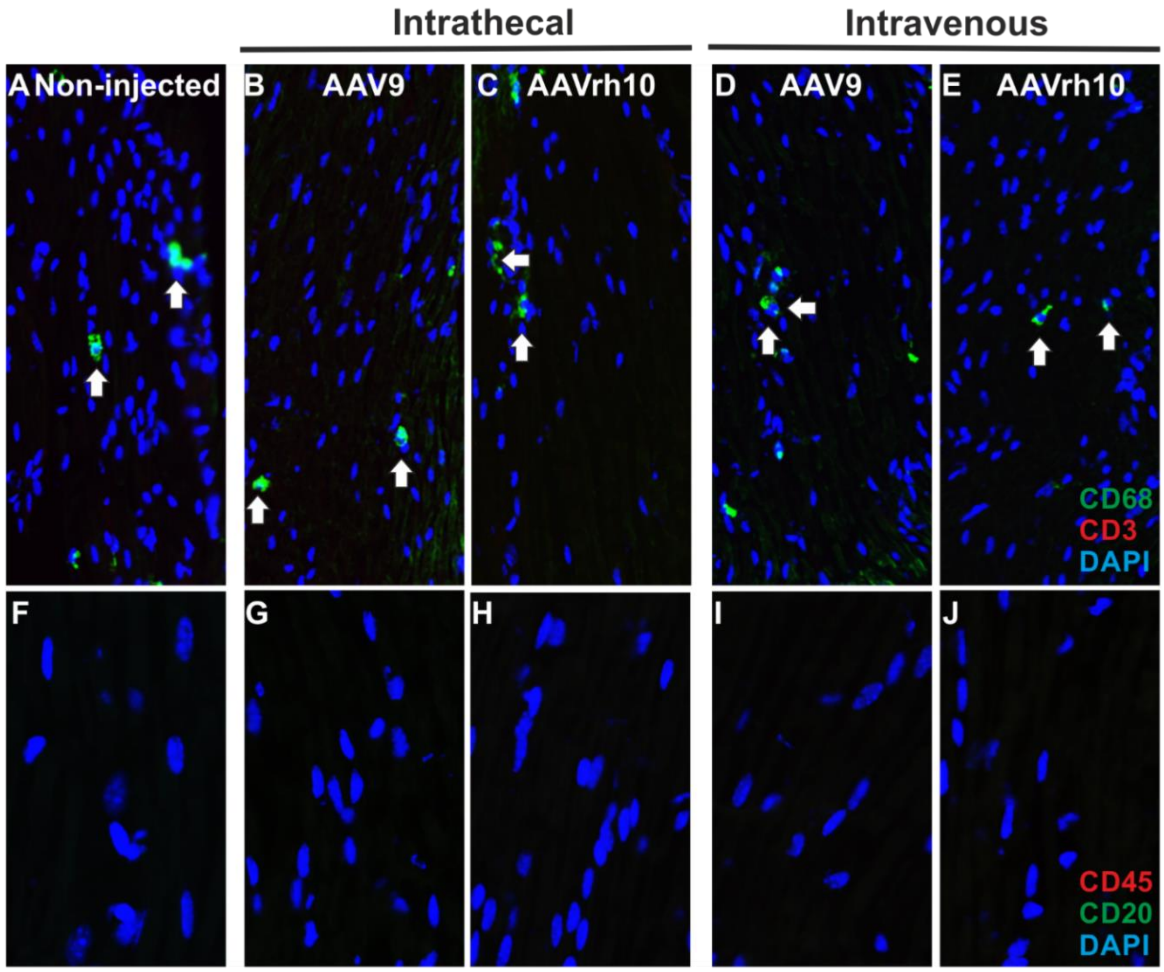

Supplementary Figure 6: Inflammatory responses in lumbar roots following intrathecal and intravenous injection of AAV9-Mpz.*GJB1* and AAVrh10-Mpz.*GJB1* vectors in *Gjb1*-null mice. CD68+ macrophages (**A-E**; arrows) were detected in both AAV9 and AAVrh10 injected mice after intrathecal (**B-C**) or intravenous (**D-E**) injection without apparent different compared to non-injected mice (**A**). CD3+, CD20+ and CD45+ cells were not detected in

tissues of mice injected with either serotype (**B-E and G-J)** or in tissues of non-injected mice (**A-F**).

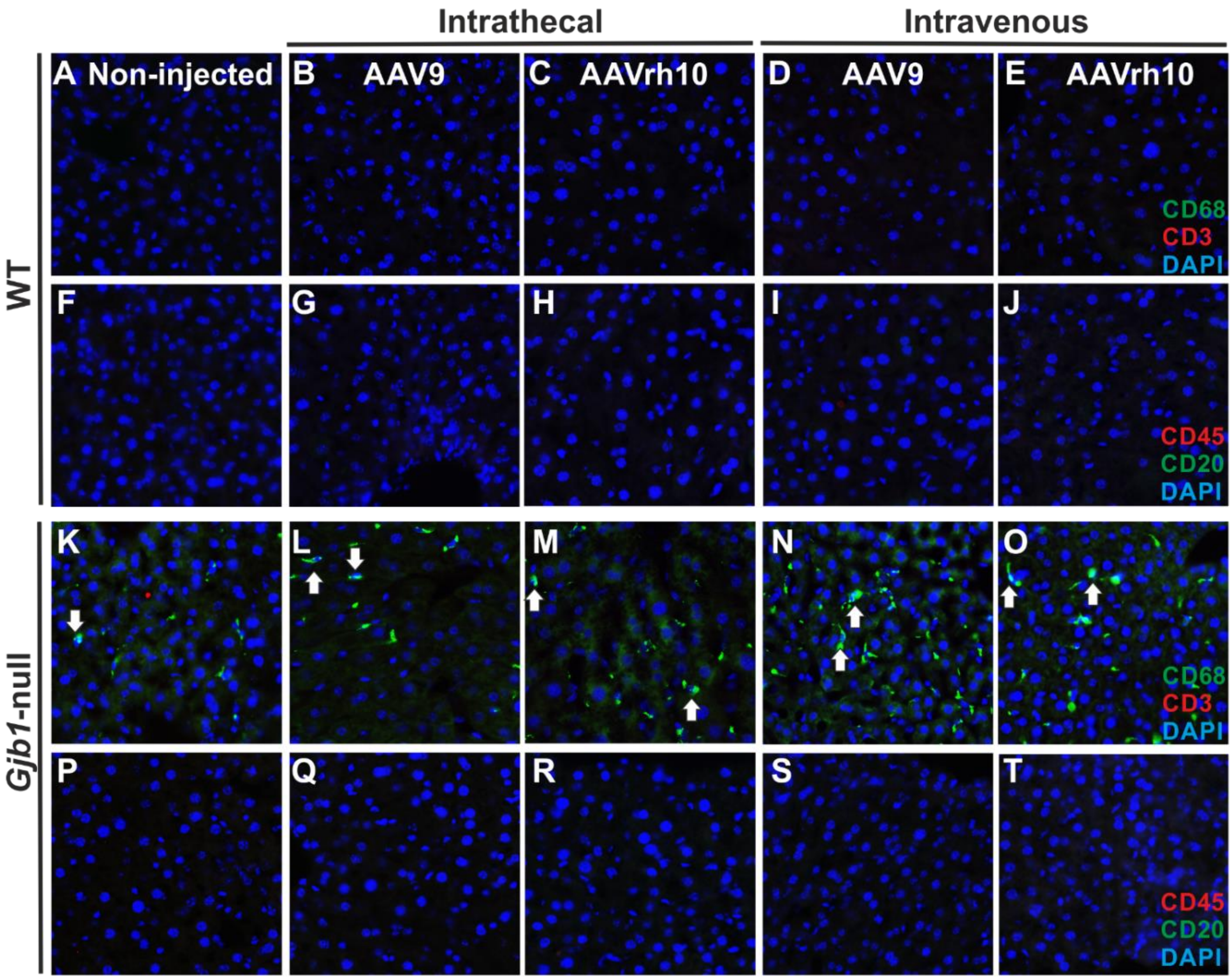

Supplementary Figure 7: Inflammatory responses in liver following intrathecal and intravenous injection of AAV9-Mpz.*Egfp* and AAVrh10-Mpz.*Egfp* vectors in WT mice and of AAV9-Mpz.*GJB1* and AAVrh10-Mpz.*GJB1* vectors in *Gjb1*-null mice. No inflammatory cells were detected in the liver of WT injected mice with both vectors similar to the non-injected mice (**A-J**). In the *Gjb1*-null injected mice (**K-T**) a number of CD68+ macrophages were detected in the liver of injected mice compared to the non-injected mice (**K-**

O) while no CD3+, CD20+ or CD45+ cells were detected in the liver of mice injected with either serotype (**K-T**).

Supplementary Table 1: Counts of inflammatory cells in PNS tissues following intrathecal or intravenous injection of AAV9-Mpz.*GJB1* or AAVrh10-Mpz.*GJB1* vectors in *Gjb1*-null mice.

	**Intrathecal injection**											
	**CD68+ AAV9-Mpz.*GJB1***	**CD68+ AAVrh10-Mpz. *GJB1***	**P value**	**CD3+ AAV9-Mpz. *GJB1***	**CD3+ AAVrh10-Mpz. *GJB1***	**P value**	**CD45+ AAV9-Mpz.*GJB1***	**CD45+ AAVrh10-Mpz.*GJB1***	**P value**	**CD20+ AAV9-Mpz.*GJB1***	**CD20+ AAVrh10-Mpz.*GJB1***	**P value**
**Lumbar roots**	8.47±1.81 (n=3)	5.48±1.38 (n=3)	>0.05	0 (n=3)	0 (n=3)	>0.05	0.03±0.03 (n=3)	0.13±0.02 (n=3)	>0.05	0 (n=3)	0 (n=3)	>0.05
**Sciatic nerve**	4.20±1.42 (n=3)	3.29±2.16 (n=3)	>0.05	0.03±0.03 (n=3)	0.39±0.20 (n=3)	>0.05	0.71±0.37 (n=3)	0.72±0.09 (n=3)	>0.05	0.06±0.06 (n=3)	0 (n=3)	>0.05
	**Intravenous injection**											
**Lumbar roots**	3.74±1.51 (n=3)	4.17±0.44 (n=3)	>0.05	0 (n=3)	0 (n=3)	>0.05	0 (n=3)	0.11±0.11 (n=3)	>0.05	0 (n=3)	0 (n=3)	>0.05
**Sciatic nerve**	2.72±0.71 (n=4)	3.43±0.87 (n=5)	>0.05	0.11±0.04 (n=4)	0.04±0.03 (n=5)	>0.05	0.83±0.13 (n=4)	0.97±0.37 (n=5)	>0.05	0 (n=4)	0 (n=5)	>0.05

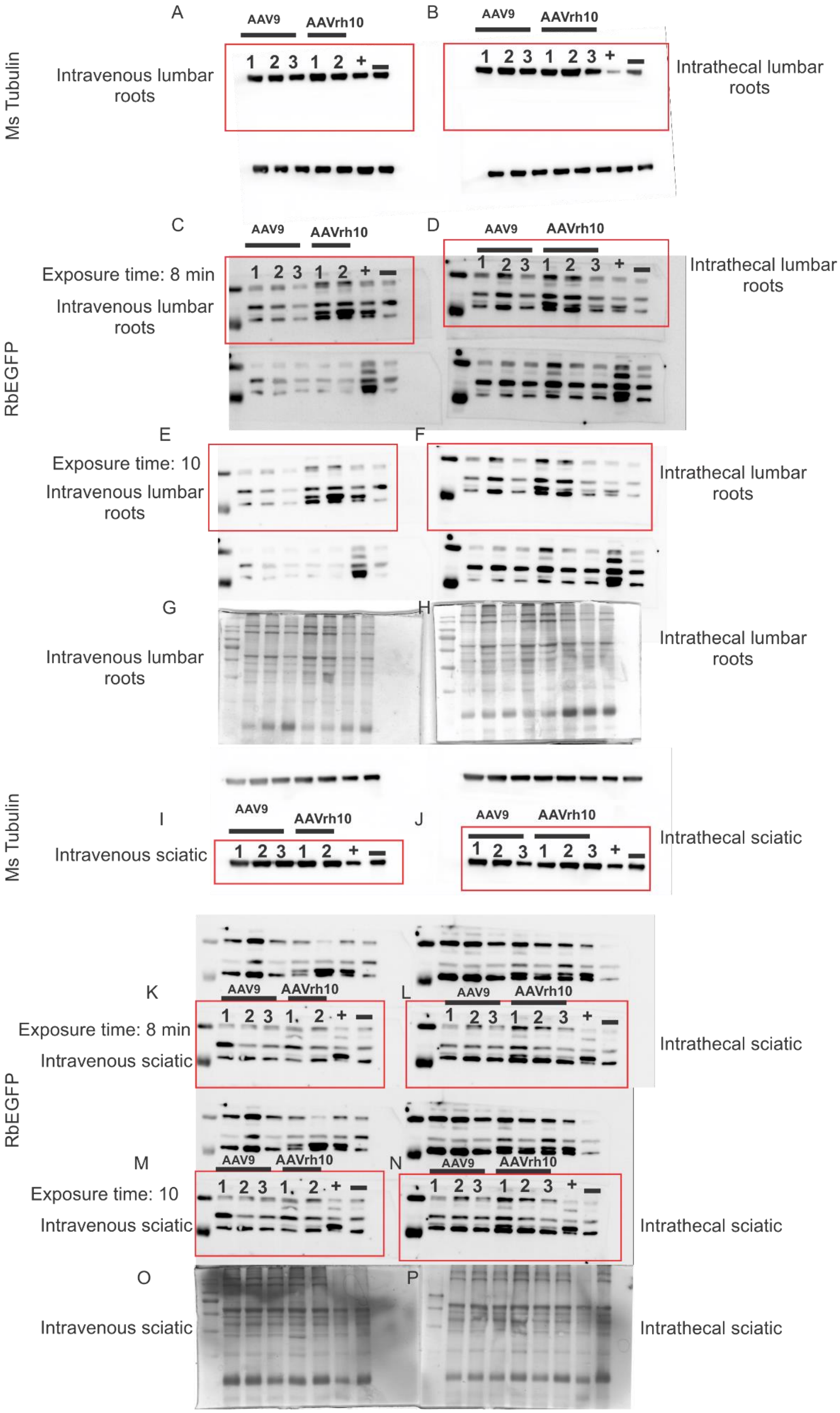
A
AAV9
AAVrh10
1 2 3 1 2 + −
Intravenous lumbar roots
B
AAV9
AAVrh10
1 2 3 1 2 3 + −
Intrathecal lumbar roots
Ms Tubulin
C
AAV9
AAVrh10
1 2 3 1 2 + −
Exposure time: 8 min
Intravenous lumbar roots
D
AAV9
AAVrh10
1 2 3 1 2 3 + −
Intrathecal lumbar roots
RbEGFP
E
Exposure time: 10
Intravenous lumbar roots
F
Intrathecal lumbar roots
G
Intravenous lumbar roots
H
Intrathecal lumbar roots
I
AAV9
AAVrh10
1 2 3 1 2 + −
Intravenous sciatic
J
AAV9
AAVrh10
1 2 3 1 2 3 + −
Intrathecal sciatic
Ms Tubulin
K
AAV9
AAVrh10
1 2 3 1 2 + −
Exposure time: 8 min
Intravenous sciatic
L
AAV9
AAVrh10
1 2 3 1 2 3 + −
Intrathecal sciatic
RbEGFP
M
AAV9
AAVrh10
1 2 3 1 2 + −
Exposure time: 10
Intravenous sciatic
N
AAV9
AAVrh10
1 2 3 1 2 3 + −
Intrathecal sciatic
O
Intravenous sciatic
P
Intrathecal sciatic

Supplementary Figure 8: Original blots and gels of immunoblot analysis of EGFP in AAV9.Mpz-*Egfp* and AAVrh10.Mpz-*Egfp* injected WT mice. EGFP expression analysis using immunoblot analysis in anterior lumbar roots after intrathecal (**D, F; corresponding to Fig. 2M**) and intravenous (**C, E; corresponding to Fig. 2O**) injection following 8 and 10 minutes of exposure. As loading control tubulin was used in both cases (**A, B; corresponding to Fig. 2M, O**). (**G, H**) Gels of root lysates of intrathecally and intravenously injected mice. EGFP expression in sciatic nerves after intrathecal (**K, M; corresponding to Fig. 2N**) and intravenous (**L, N; corresponding to Fig. 2P**) injection following 8 and 10 minutes of exposure. Tubulin was used as loading control in both cases (**I, J; corresponding to Fig. 2N, P**). (**O, P**) Gels of sciatic nerve lysates of intrathecally and intravenously injected mice.

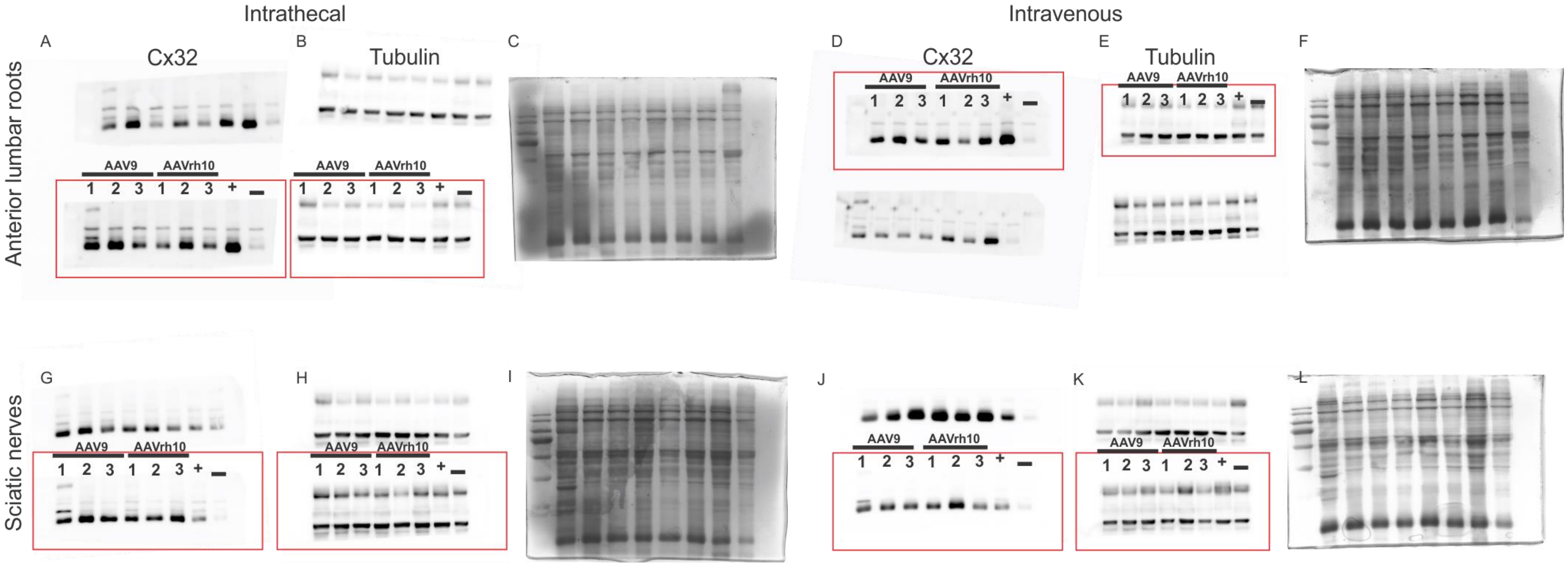

Supplementary Figure 9: Original blots and gels of immunoblot analysis of Cx32 in AAV9.Mpz-*GJB1* and AAVrh10.Mpz-*GJB1* injected *Gjb1*-null mice. Cx32 expression analysis using immunoblot analysis in anterior lumbar roots after intrathecal (**A; corresponding to Fig. 3M**) and intravenous (**D; corresponding to Fig. 3O**) injection. As loading control tubulin was used in both cases (**B, E; corresponding to Fig. 3M, O**). Cx32 expression in sciatic nerves after intrathecal (**G; corresponding to Fig. 3N**) and intravenous (**J; corresponding to Fig. 3P**) injection. Tubulin was used as loading control in both cases (**H, K; corresponding to Fig. 3N, P**). (**C, F**) Gels of root lysates of intrathecally and intravenously injected mice. (**I, L**) Gels of sciatic nerve lysates of intrathecally and intravenously injected mice.
